# Using Landsat Satellite Imagery to Investigate Spatial and Temporal Variation in Life History Traits in a Long‐Term Study Population of Superb Fairy‐Wrens *Malurus cyaneus*

**DOI:** 10.1002/ece3.73852

**Published:** 2026-06-14

**Authors:** Richard S. Turner, Ophélie J. D. Lasne, Lei Lv, Helen L. Osmond, Andrew Cockburn, Loeske E. B. Kruuk, Kara N. Youngentob

**Affiliations:** ^1^ Division of Ecology & Evolution, Research School of Biology Australian National University Canberra Australian Capital Territory Australia; ^2^ Institute of Ecology & Evolution, School of Biological Sciences University of Edinburgh Edinburgh UK; ^3^ School of Ecology Hainan University Haikou China; ^4^ The Fenner School of Environment & Society Australian National University Canberra Australian Capital Territory Australia

**Keywords:** avian life histories, climate change, Landsat, *Malurus cyaneus*, NDVI, satellite imagery, vegetation productivity

## Abstract

Long‐term, individual‐level studies can provide valuable insights into the effects of climate and landscape change on the ecology and population dynamics of wild animals. However, many such studies lack environmental data collected at the spatial and temporal resolutions needed to determine how populations respond to changing conditions. In these cases, the retrospective use of satellite‐derived data can provide a way to recover past environmental information. Using a 27‐year dataset of an Australian insectivorous passerine, the superb fairy‐wren *Malurus cyaneus* , we assessed how climate variation influences vegetation productivity and, indirectly, superb fairy‐wren life history traits through potential changes in trophic interactions. Specifically, we combined long‐term, individual‐level monitoring of superb fairy‐wrens and local weather records with Landsat satellite imagery, from which we derived measures of vegetation productivity using the Normalised Difference Vegetation Index (NDVI) as a proxy for food availability through arthropod abundance. We found a complex set of associations between NDVI and different components of weather, when considering both concurrent and lagged effects. Our analyses of the causes of seasonal variation in superb fairy‐wren life history traits demonstrated that NDVI was associated with: (i) temporal variation in breeding success, with years with high spring and summer NDVI values having relatively high average breeding success; and (ii) spatial variation in adult mortality in autumn and winter, with superb fairy‐wren territories with low autumn–winter NDVI values having higher average mortality rates. Notably, autumn–winter NDVI values were found to have remained relatively consistent over time, indicating that vegetation productivity cannot explain recently observed increases in adult autumn–winter mortality. Our study illustrates the potential of using long‐term Landsat satellite imagery to investigate whether associations between animal life history traits and climate are mediated by vegetation productivity and to what extent temporal trends are influenced by climate change.

## Graphical abstract

We used 27 years of data from an insectivorous passerine in southeastern Australia, the superb fairy‐wren *Malurus cyaneus* , to assess how climate variation influences vegetation productivity and, indirectly, reproduction and survival via potential trophic interactions. Specifically, we integrated long‐term, individual‐level monitoring data with local weather records and Landsat satellite imagery. From the satellite data, we derived measures of vegetation productivity using the Normalised Difference Vegetation Index (NDVI), which we used as a proxy for food availability through its association with arthropod abundance. We found that NDVI was associated with: (i) temporal variation in breeding success, with years with high spring and summer NDVI values having relatively high average breeding success; and (ii) spatial variation in adult mortality in autumn and winter, with superb fairy‐wren territories with low autumn–winter NDVI values having higher average mortality rates. Overall, our study illustrates the potential of using long‐term Landsat satellite imagery to investigate whether associations between animal life history traits and climate are mediated by vegetation productivity and to what extent temporal trends are influenced by climate change.

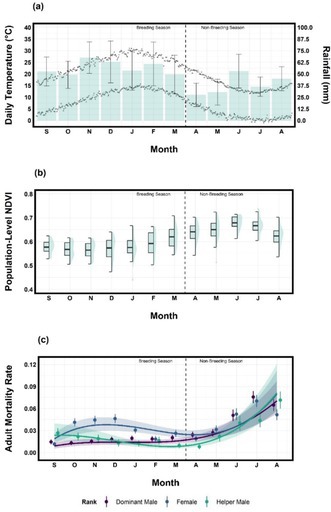

## 1 Introduction

Global climate change—including warming temperatures and altered precipitation patterns—is affecting the vegetation dynamics of ecosystems worldwide (Hoffmann et al. 2018; Nemani et al. 2003; Parmesan and Yohe 2003; Walther et al. 2002; Zhao and Running 2010), with widespread changes in plant growth, phenology, distribution, and abundance having already been observed (Cleland et al. 2007; Hoffmann et al. 2018; Nemani et al. 2003; Piao et al. 2019; van der Putten et al. 2010; Zhao and Running 2010). Because vegetation underpins primary productivity, these shifts can influence trophic interactions by altering the quality, abundance, and accessibility of food and other structural habitat resources for higher‐order consumers (Butt et al. 2026; Cole et al. 2015; Dubos et al. 2018; Pettorelli et al. 2005, 2006, 2007; Gardner et al. 2014). Consequently, climate‐driven changes in vegetation productivity can directly influence wild animal population dynamics by affecting key life history traits such as reproduction and survival.

Despite its potential significance, few long‐term animal studies have incorporated vegetation productivity data into their analyses. This may be attributed to difficulties in manually collecting the necessary information on vegetation at the appropriate spatial and temporal scales (Block et al. 1987; Gotfryd and Hansell 1985; Johnson 2007), or that the collection of such data was not originally a priority in these studies. Consequently, researchers have frequently relied on climate‐related variables as indirect proxies for vegetation productivity and, subsequently, food availability. For example, observed declines in body size in association with warming temperatures are typically interpreted as evidence of climate‐driven changes in vegetation productivity leading to a decline in food over time (Bailey et al. 2020; Gardner et al. 2014; Kruuk et al. 2015; Sanz et al. 2003). However, without vegetation‐based assessments to demonstrate that these life history traits are directly influenced by changes in vegetation productivity or the ability to link climate data to vegetation productivity data, it remains challenging to identify causal explanations, or to differentiate between climate‐driven changes to vegetation productivity and those caused by other factors such as pollution, land use changes, or the spread of invasive species or diseases (Jiang et al. 2017; Laliberté and Tylianakis 2012; Maitima et al. 2009; Vilà et al. 2011; Yue and Unger 2018).

One potential solution to this problem is to use remotely sensed satellite data to retrospectively recover information about the past vegetation properties of environments (Anderson et al. 2010; Fokeng and Fogwe 2022; Neigh et al. 2008; Pettorelli et al. 2014; Turner et al. 2003). Among the different vegetation indices that can be derived from satellite data (Bannari et al. 1995), the most commonly used is the Normalised Difference Vegetation Index (NDVI; Pettorelli 2013; Rouse et al. 1974), which is widely recognised as an accurate and reliable indicator of vegetation productivity (Butt et al. 2026; Nemani et al. 2003; Pettorelli 2013).

In recent decades, there has been a substantial rise in interest among ecologists in utilising satellite data—and, in particular, measures of NDVI—to assess the interactions and responses of wild animal populations to environmental change. This interest is driven, in part, by the increasing accessibility of pre‐processed datasets available on a global scale that have undergone some degree of quality control. To date, most studies investigating associations between satellite‐derived NDVI measures and wild animal populations have focused on the population‐level, exploring relationships between NDVI and the abundance, richness, and distribution of herbivorous species (Hasik et al. 2025; Hurley et al. 2014; Kotzur et al. 2024; Pettorelli et al. 2005, 2006, 2007, 2011; Youngentob et al. 2015). However, recent research has shown that these data can be used successfully to explore ecological questions in omnivorous and non‐herbivorous species from higher‐order trophic levels (Cole et al. 2015; Gardner et al. 2014; Hurley et al. 2014; Pettorelli et al. 2011; Saino et al. 2004; Sanz et al. 2003; Smith et al. 2017; Willems et al. 2009). For example, Cole et al. (2015) found that NDVI could accurately predict the abundance of winter moth larvae *Operophtera brumata* , which serves as an important food source for great tits *Parus major* and blue tits *Cyanistes caeruleus* . Additionally, they found that NDVI could also subsequently predict the reproductive phenology of both bird species (Cole et al. 2015).

The increasing availability of fine spatial and temporal resolution satellite data also offers potential for integrating these data into long‐term, individual‐level studies, an area of research which is yet to be comprehensively explored. By combining satellite‐derived NDVI estimates with information acquired from such studies, an opportunity arises to gain novel insights into the associations between climate change and variation in individual life histories. This integrated approach also provides a retrospective means to examine whether the impacts of vegetation productivity, and hence food availability, can mediate these associations (McLean et al. 2022; Sheldon et al. 2022). In this study, we investigated (i) the impact of climate on vegetation productivity and (ii) whether climate‐driven changes in vegetation productivity affect the mortality and breeding success of a wild population of superb fairy‐wrens *Malurus cyaneus* , using 27 years of local weather data, Landsat satellite‐derived NDVI data, and year‐round individual‐level bird census data.

### 1.1 Target Species

Superb fairy‐wrens are small passerines (*c*. 10 g; Dunning Jr 2007) endemic to southeastern Australia (Cockburn et al. 2016). They are insectivorous and typically forage in open grassy areas, among leaf‐litter, and from the lower branches of vegetation (Rowley 1965; Rowley and Russell 1997). Although their diet consists predominantly of arthropods, the inspection of stomach contents has also revealed the presence of seeds, primarily from *Rhagodia* spp. (Rowley 1965). Superb fairy‐wrens are facultative cooperative breeders, and a territory held by a dominant, socially monogamous pair may also contain up to five ‘helpers’ (Cockburn, Sims, et al. 2008; Hajduk et al. 2021). These ‘helpers’ are always males, as they are philopatric and generally live and die on either their natal territory or an immediate neighbouring territory (Mulder 1995). Male dominance ranking on a territory is determined by age. When the dominant male dies, the oldest helper male residing on the territory takes the dominant position (Cockburn, Sims, et al. 2008). In contrast, female superb fairy‐wrens disperse to a new territory as juveniles, often moving over long distances. They become reproductive adults in their first breeding season post‐fledging and thereafter rarely move territories (Cockburn et al. 2003; Mulder 1995). While breeding females are solely responsible for nest‐building and incubation, all group members defend and provision the brood (Dunn et al. 1995; Dunn and Cockburn 1996; Yasukawa and Cockburn 2009).

Previous research has investigated various aspects of the complex life history of superb fairy‐wrens in relation to climate and climate change. Specifically, findings from our long‐term study population have shown that adult superb fairy‐wrens have higher mortality during autumn and winter in years when temperatures are warmer (Lv et al. 2023). Additionally, drier and colder conditions can lead to a delay in male superb fairy‐wrens moulting into their nuptial plumage, and anecdotal observations suggest that moulting males may also be more susceptible to dying following sudden transitions from warm and wet conditions to heavy frosts (Cockburn, Osmond, and Double 2008; Lv et al. 2023; van de Pol et al. 2012).

Warmer and drier conditions during the breeding season in spring and summer have also been associated with larger eggs laid by female superb fairy‐wrens (Langmore et al. 2016), reduced average body mass of nestlings (Kruuk et al. 2015), and earlier cessation of the breeding season (Lv et al. 2019). Conversely, wetter conditions have been associated with increased breeding success (i.e., the total number of offspring to reach independence; Cockburn, Osmond, and Double 2008). While these studies indicate negative effects of climate warming and more variable rainfall patterns on superb fairy‐wren life histories, it remains unclear whether these effects are direct (e.g., by affecting individuals' thermal physiology) or indirect (e.g., by altering vegetation productivity and hence influencing food availability through changes in arthropod abundance).

Recent findings have additionally shown that fine‐scale spatial variation in structural vegetation characteristics can influence superb fairy‐wren breeding behaviour and performance (Backhouse et al. 2023; Turner et al. 2023). However, these measurements are only available for a single year, and, as yet, we lack a comprehensive understanding of the relative importance of this variation in vegetation compared to climate in shaping the life histories of superb fairy‐wrens. The availability of long‐term Landsat satellite‐derived NDVI data therefore provides an excellent opportunity to evaluate the relative associations of these variables by providing retrospective measures of aspects of vegetation across our study area. The aims of this study were as follows:

– To identify any temporal trends in climate and vegetation productivity for the study area over the 27 years of the study, asking: Do climate and/or NDVI parameters change over time?
– To determine the relevant timing and nature of any effects of climate on vegetation productivity during the study period, asking: How does NDVI change in response to current or previous weather conditions?
– To test whether spatial or temporal variation in vegetation productivity, as a proxy for variation in food abundance, is associated with superb fairy‐wren life history traits. Here, we compare the effects of population‐level (temporal) and relative territory‐level (spatial) NDVI on adult mortality and breeding success, asking: How do these local and landscape‐scale vegetation dynamics relate to variation in survival and reproductive outcomes?

## 2 Materials and Methods

### 2.1 Study Area

The study area is located in Canberra, Australian Capital Territory, Australia (35°16′30.0″S, 149°06′28.8″E) and encompasses an area of *c*. 65 ha that includes a managed area (*c*. 43 ha) of native Australian plants in the Australian National Botanic Gardens (ANBG) and an unmanaged area (*c*. 22 ha), which is part of the adjacent Black Mountain Nature Reserve. The vegetation in the managed area receives some supplementary irrigation. Details of the vegetation structure and composition in the study area have been described elsewhere (Backhouse et al. 2023; Fraser and Purdie 2020; Turner et al. 2023). In brief, the study area is broadly characterised as mature open sclerophyll forest, with evergreen *Eucalyptus macrorhyncha* and *E. rossii* as the primary tree species, and *Acacia* spp., *Callistemon* spp., *Notodanthonia* spp., *Rytidosperma palladium*, *Triodia scariosa*, and *Lomandra longifolia* as the dominant understorey shrubs and grasses. The managed area also includes three semi‐artificial habitats (specifically a ‘rainforest’ area, a ‘desert’ area, and a grass lawn). We have shown previously that superb fairy‐wrens do not inhabit these areas of the study area (Backhouse et al. 2023) and so, for the purpose of this study, we do not use data from these areas in our analyses.

**FIGURE 1:**
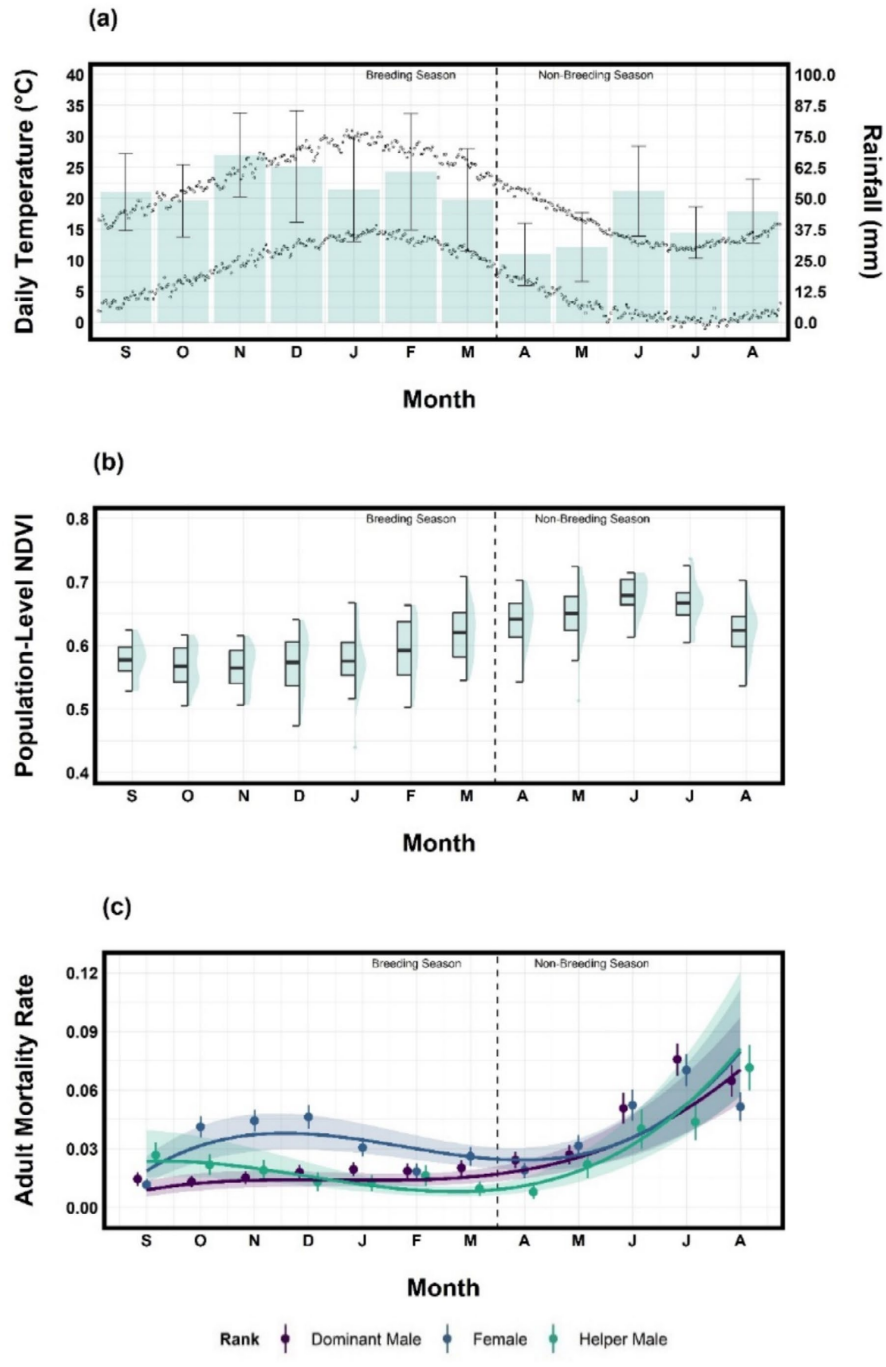
Changes in (a) weather conditions, (b) NDVI, and (c) the mortality rate of adult superb fairy‐wrens in each month throughout the year, beginning from September to August. In panel (a) the points show the mean daily maximum temperature (°C) and mean daily minimum temperature (°C) of each Julian date from 1 September 1994 to 31 August 2021 (left‐hand *y* axis), and the bars show the mean (±SE) monthly rainfall (mm, right‐hand *y* axis). In panel (b) the boxes show the monthly population‐level NDVI (plus upper and lower quartiles); the whiskers show the ±95% confidence intervals; and the histograms show the distribution of the population‐level NDVI values in each month across the years of the study. In panel (c) the regression lines show the model estimated marginal means (±95% confidence intervals) of monthly adult mortality rates of individuals of different sex and rank in each territory: Female (blue); dominant male (purple); and helper male (green). Model estimates were taken from the monthly resolution model that contained all months within a given year (provided in Table S1) and are corrected for fixed effect parameters. The points show the mean (±SE) of the raw data for each sex and rank per month. In all panels the dashed line indicates the cut‐off point between the respective periods considered as the breeding season (i.e., spring–summer period from September to March) and the non‐breeding season (i.e., autumn–winter period from April to August).

Located in the Southern Tablelands of New South Wales (on an inland plateau *c*. 650 m above sea level), the region has a seasonal continental climate, with coldest temperatures in July and hottest in January (Figure 1; Fraser and Purdie 2020; Lv et al. 2023). Moderate rainfall occurs throughout the year, peaking slightly in November due to summer thunderstorms. Total annual rainfall is highly variable from year to year and the region has experienced several years of drought during the study period, with the most notable occurring between 2001 and 2009 (known as the Millennium Drought; van Dijk et al. 2013) and from 2017 to 2019 (Australian Bureau of Meteorology, https://www.bom.gov.au/climate/data).

### 2.2 Meteorological Data

We obtained daily weather data for Canberra airport, located approximately 8 km east of the study area, from the Australian Bureau of Meteorology (https://www.bom.gov.au/climate/data). Using these data, we calculated the following weather parameters for each spring–summer breeding season and autumn–winter non‐breeding season period in each year:

– *Maximum temperature*: The mean of all daily maximum temperatures recorded during a given spring–summer and autumn–winter period in each year.
– *Minimum temperature*: The mean of all daily minimum temperatures recorded during a given spring–summer and autumn–winter period in each year.
– *Rainfall*: The total rainfall recorded during a given spring–summer and autumn–winter period in each year.

### 2.3 Landsat Imagery Data

Landsat imagery provides a long‐term record of Earth surface reflectance from a series of multispectral Earth‐observation satellites. Since the launch of Landsat Thematic Mapper in 1984, Landsat data have provided near‐global coverage at a 30 m spatial resolution and an approximate 16‐day revisit interval, making them well suited for examining spatial and temporal variation in vegetation productivity using indices such as NDVI (Wulder et al. 2019; Zhu et al. 2019). For this study, we acquired analysis‐ready Landsat imagery data (ARD) from Digital Earth Australia's (DEA) Open Data Cube (Dhu et al. 2017; Lewis et al. 2017). This data consisted of Landsat 5 (Thematic Mapper [TM]), Landsat 7 (Enhanced Thematic Mapper Plus [ETM+]), and Landsat 8 (Operational Land Imager and Thermal Infra‐Red Scanner [OLI‐TIRS]) imagery.

The Landsat ARD images provided by DEA had undergone pre‐processing to normalise surface reflectance, correct for terrain illumination in sloped areas, and adjust to a single nadir view with a solar zenith angle of 45°, resulting in normalised Bidirectional Reflectance Distribution Function (BRDF)‐adjusted reflectance (NBAR) (Lewis et al. 2017). Additionally, each image included pixel‐level observation attributes (OA) that categorised each pixel as one of the following: no data, valid data, cloud, shadow, snow, or water. These categories were determined using the automated *function of mask* (fmask) algorithm (Zhu and Woodcock 2012). To ensure consistency with our superb fairy‐wren territory polygons, we obtained the Landsat ARD in spatial reference Geocentric Datum of Australia 1994, Map Grid of Australia Zone 55 (Collier and Steed 2001).

We obtained Landsat 5 images from 1 September 1994 to 31 December 2011 and Landsat 8 images from 1 January 2013 to 31 August 2021. For both datasets, we ensured images contained less than 5% cloud cover or non‐valid data, as these attributes can influence reflectance values from the spectral bands in the images (Zhu and Woodcock 2012). Regarding Landsat 7 images, which we obtained from 1 January 2012 to 31 December 2012, we used a slightly different approach due to a known issue with the Scan Line Corrector (SLC) on its ETM+ instrument. In May 2003, the SLC experienced a failure, resulting in data gaps of approximately 22% in the acquired images (Storey et al. 2005; Zeng et al. 2013). As a result, we obtained Landsat 7 images with < 20% cloud cover or non‐valid data. Although the SLC‐off images have these data gaps, the pixels outside these gaps have maintained their radiometric and geometric quality from before the SLC failure (Storey et al. 2005; Zeng et al. 2013). Nevertheless, we also conducted all analyses without using data from Landsat 7, and these analyses yielded effectively identical results (which we do not present here).

#### 2.3.1 Processing Landsat Imagery Data and Calculating NDVI

To obtain NDVI values, we performed pixel‐level processing on each Landsat ARD image as follows: First, we used the pixel‐level OA to identify and exclude non‐valid pixels, including those categorised as no data, cloud, shadow, snow, or water. This step ensured that only valid pixels were retained. Second, using QGIS (v.3.16.7; QGIS.org 2022), we extracted spatial boundaries of anthropogenic attributes present in our study area, such as buildings, carparks, and roads, which we derived from a Google Satellite Hybrid map. We then removed all pixels from each Landsat ARD image that overlapped with these impervious surfaces. This step allowed us to focus exclusively on vegetation‐only pixels. Third, after excluding non‐valid and artificial attribute pixels, we calculated NDVI for the remaining pixels using the following formula:

(1) $$\text{NDVI}=\frac{\left(\mathrm{NIR}-\mathrm{Red}\right)}{\left(\mathrm{NIR}+\mathrm{Red}\right)}$$

where ‘NIR’ and ‘Red’ are the respective wavelength values of the near‐infrared and red spectral bands captured by the satellite's optical sensor (Rouse et al. 1974). NDVI values range from −1.0 to +1.0, with negative values and low positive values being indicative of non‐vegetation features such as water, bare soil, rock, asphalt, cloud, shadow, and buildings (Neigh et al. 2008), and larger positive values being indicative of increasingly greener and hence more productive vegetation (Pettorelli 2013).

For the purpose of this study, we limited our analyses to pixels containing NDVI values ranging from 0.15 to 0.85. The lower limit allowed us to avoid pixels containing only a small fraction of vegetation, whereas the upper limit was set to avoid potential biases from a saturation effect that can occur in very dense vegetation areas. In such cases, the reflectance values of the NIR and Red bands can become extremely high, resulting in truncated or saturated NDVI values (Mutanga et al. 2023). Our dataset therefore consisted of NDVI values from a total of 382,117 pixels across 502 images spanning our 27‐year study period. The average time interval between each image was 20.1 ± 20.0 days (mean ± SD; range: 1–192 days).

### 2.4 Superb Fairy‐Wren Data

#### 2.4.1 Study Population

We used uninterrupted, year‐round census data collected on the population of superb fairy‐wrens in the study area from 1 September 1994 to 31 August 2021. Almost all individuals in the study population were uniquely colour‐banded and individually recognisable. Throughout the study period, the study area supported between 34 and 89 superb fairy‐wren territories each year, ranging from 0.07 to 6.49 ha in size (mean territory size ± SD: 0.95 ± 0.58 ha, *n* = 1864 territory‐years). Details of the processes used to measure each superb fairy‐wren territory are described below.

The breeding season of the superb fairy‐wren spans the austral spring and summer, typically starting around September and lasting until March, with a peak in activity in December (Lv et al. 2019). In this study, we defined the breeding season as the period of time from 1 September to 31 March. The non‐breeding season was therefore considered to encompass the austral autumn and winter, from 1 April to 31 August. Throughout this study, the term ‘year’ is used to refer to a 12‐month period commencing on 1 September, to correspond with the onset of the superb fairy‐wren breeding cycle. For example, the year ‘1994’ represents the time period from 1 September 1994 to 31 August 1995, and so forth.

#### 2.4.2 Measuring Superb Fairy‐Wren Territory Boundaries

The processes used to measure each superb fairy‐wren territory are comprehensively described in Backhouse et al. (2023). In brief: territory boundaries were determined through field observations of aggressive encounters between individuals residing on neighbouring territories (Cockburn et al. 2003). Each territory boundary was initially hand‐drawn on a 30 m grid map of the study area, using a series of connected vertices. Recognisable physical landmarks in the study area (e.g., paths, buildings, and specific landscaping features) were used to determine the position of each vertex, accurate to one decimal point fraction (i.e., within a 9 m^2^ grid cell).

Territory boundaries were monitored multiple times per year. The hand‐drawn maps that were closest to 15 November were scanned, imported into ArcGIS (v.10.3.1; ESRI Inc., Redlands, CA, USA), and then digitised (Backhouse et al. 2023). This particular date aligns with a period when group dynamics and territory boundaries are typically established for that year, although boundaries may be temporally relaxed during the non‐breeding season (A. Cockburn, unpublished data). Spatial polygons of each superb fairy‐wren territory were assigned a territory ID. Often there was very little change in territory boundaries from one year to the next. In such cases, territories in the same location in consecutive years were given the same territory ID. However, when there were changes in their boundaries compared to the previous year, territories were assigned new territory IDs (Backhouse et al. 2023). The territory polygons were projected in spatial reference Geocentric Datum of Australia 1994, Map Grid of Australia Zone 55 (Collier and Steed 2001). All analyses presented here were subsequently conducted using this projection.

#### 2.4.3 Measuring Mortality Rate

All individuals in the study population were routinely censused throughout each year (Lv et al. 2023). We aimed to observe each individual at least three times per week during the breeding season and at least once per week in the non‐breeding season. We considered an individual as having died if they were not observed during three consecutive censuses or at any point thereafter. We estimated an individual's date of death as the midpoint between the date when they were last observed and the date of the first census on which they were not observed. Because it is not possible to distinguish mortality from dispersal outside the study area in juvenile female superb fairy‐wrens, we focused our analyses on adult birds only (following, e.g., Lv et al. 2023). Note, adults in their first breeding season post‐fledging were treated as being one‐year old, though, in actuality, they may vary in age from 6 months to one‐year old due to differences in their hatch‐date.

During the study period, there was a total of 64 months (19.75% of the total) where Landsat ARD images were not usable. This data gap was primarily attributed to the presence of high cloud cover on the days when the satellite passed over the study area (details below). Given this gap, we therefore focused on analysing adult mortality rates during the breeding and non‐breeding seasons at a seasonal spring–summer and autumn–winter resolution. However, for completeness, we also repeated all analyses at a monthly and annual resolution. The results from these additional analyses are presented separately as Tables S1 and S2. It is important to note that none of the Landsat ARD images were usable for the entirety of the 2007 non‐breeding season. Consequently, all analyses involving non‐breeding season observations were based on 26 rather than 27 years of data.

#### 2.4.4 Measuring Breeding Success

Superb fairy‐wrens are multi‐brooded: during the breeding season a female can initiate as many as ten clutches and successfully rear up to four broods. However, rates of nest predation are high and often only one brood (if any) successfully reaches independence (Lv et al. 2019; Turner et al. 2023). Clutch sizes can range from one to five eggs but clutches with three eggs are most common (Cockburn et al. 2016; Rowley and Russell 1997). We used the total number of offspring to reach independence as a measure of breeding success each year, with independence defined as 4 weeks post‐fledging (following, e.g., Cockburn, Sims, et al. 2008; Hajduk et al. 2018). Although most offspring are still being cared for at this age, the earliest known age of dispersal in the study area is 5 weeks post‐fledging; this 4‐week cut‐off point therefore avoids any confusion between dispersal and mortality (Hajduk et al. 2018). Determining associations between male breeding success and local environmental conditions is challenging due to exceptionally high rates of extra‐pair paternity and the fact that males may sire offspring in multiple territories across the study area (Cooper et al. 2020; Hajduk et al. 2018, 2021). Therefore, in this study, we focused solely on female breeding success (following, e.g., Lv et al. 2019).

### 2.5 Combining NDVI and Superb Fairy‐Wren Datasets

We used the *join attributes by location* algorithm in QGIS (v.3.16.7; QGIS.org 2022) to superimpose and combine the NDVI values from the 382,117 pixels onto the spatial polygons representing each superb fairy‐wren territory, grouping both datasets by each year of the study. As the superb fairy‐wren territories are irregular in their shapes and sizes, we assigned pixels to specific territories based on their spatial relationship as follows: If a pixel touched or partially fell within the boundaries of a given territory, we assigned it to that territory (QGIS.org 2022). Thus, due to the 30 × 30 m size of each pixel, the NDVI values from a given pixel for each Landsat ARD image were often assigned to multiple territories.

#### 2.5.1 Measuring Territory‐Level and Population‐Level NDVI

From this combined dataset, we derived two seasonal NDVI measurements for each spring–summer breeding season and autumn–winter non‐breeding season in each year:

– Mean *territory‐level NDVI*: This measurement was determined by averaging all NDVI values from all pixels within a specific superb fairy‐wren territory during a given breeding and non‐breeding season in each year.
– Mean *population‐level NDVI*: This measurement was determined by averaging all NDVI values from all pixels across all superb fairy‐wren territories during a given breeding and non‐breeding season in each year.

To distinguish between the spatial and temporal effects of NDVI in our analyses of superb fairy‐wren adult mortality and breeding success, we employed a mean‐centring approach commonly used to examine variation between and within individuals (van de Pol and Verhulst 2006; van de Pol and Wright 2009). This approach involved standardising the territory‐level NDVI values by subtracting the corresponding population‐level NDVI value. By doing so, we established the *territory‐level relative‐NDVI*, which measures the deviation of NDVI within each territory compared to the overall population‐level average during the respective season. For analyses presented in Tables S1 and S2, we derived these measurements at a monthly and annual resolution, respectively.

### 2.6 Statistical Analysis

Analyses were conducted using a Bayesian framework implemented in the package ‘brms’ (v.2.15.0; Bürkner 2017) in R (v.4.0.5; R Core Team 2021). Explanatory parameters were mean standardised prior to analysis to allow for effect size comparisons (Harrison et al. 2018; Schielzeth 2010).

#### 2.6.1 Temporal Trends in Weather Conditions and Population‐Level NDVI

We fitted a total of eight Bayesian Generalised Additive Models (GAMs; with Gaussian‐error distributions) to test whether any of the three weather parameters or the population‐level NDVI had changed directionally across the study period during spring–summer and autumn–winter. Each model included a fixed effect of year (fitted using 10 knot penalised cubic regression splines; Wood and Augustin 2002). All models were fitted on 4 independent Markov Chain Monte Carlo (MCMC) chains for 15,000 iterations, with a thinning interval of 20 and a warm‐up period of 5000 iterations (resulting in 2000 posterior samples), specifying default priors (Bürkner 2017).

#### 2.6.2 Identifying Effects of Weather on Population‐Level NDVI

To account for other seasonal effects on population‐level NDVI during spring–summer and autumn–winter, we first fitted a total of eight Bayesian linear regression models of population‐level NDVI without any weather parameters. These models separately contained either linear or non‐linear (i.e., quadratic, cubic, or quartic) fixed effects of year (fitted as a continuous covariate). Further details are provided as Appendix S1: Methods.

We used the Bayesian log predictive density leave‐one‐out (ELPD_LOO_) method to compare the predictive performance of these models (Vehtari et al. 2017, 2020). The results from this comparison indicated that all models in each analysis demonstrated similar predictive performance (Appendix S1: Methods; Table S3). Therefore, we selected the most parsimonious models, which included only a linear effect of year, as the initial baseline for our spring–summer and autumn–winter population‐level NDVI analyses.

Building on these baseline models, we considered: (i) the effects of maximum temperature, minimum temperature, and rainfall separately; (ii) additive effects of maximum temperature and rainfall, as well as minimum temperature and rainfall; and (iii) interactions between maximum temperature and rainfall, and between minimum temperature and rainfall. Weather data from the same season as the population‐level NDVI value and the preceding season were tested. Note, due to their high correlation, maximum temperature and minimum temperature were not included in the same models. As before, we used ELPD_LOO_ to identify the top‐ranked model for each analysis (Vehtari et al. 2017, 2020; Table S4). Because of the large number of models being considered, we used a randomisation approach in order to assess the possibility of false positive results (i.e., results occurring by chance). This approach involved comparing the observed ELPD_LOO_ value from the top‐ranked model to 100 ELPD_LOO_ values from models using randomised data. A given top‐ranked model was deemed reliable if its ELPD_LOO_ value fell outside the distribution of ELPD_LOO_ values from the 100 models using randomised data (i.e., *p*(ELPD_LOO_) < 0.01; Figure S1). If a false positive occurred, we report the result for completeness but do not consider it further. Each model was fitted on four independent MCMC chains for 15,000 iterations, with a thinning interval of 20 and a warm‐up period of 5000 iterations (resulting in 2000 posterior samples), specifying weakly informative priors with a normal error distribution (*μ*: 0; *σ* ^2^: 1; Gelman et al. 2015).

#### 2.6.3 Seasonal Adult Mortality Analyses

We fitted a Bayesian hierarchical generalised linear regression model (with Bernoulli‐error distribution and logit‐link function) to assess relationships between vegetation productivity and adult mortality during the spring–summer breeding season and the autumn–winter non‐breeding season. Each model included the following three random effects, which were treated as multi‐level factors:

– *Year*: To account for multiple measurements within each year.
– *Bird ID*: To account for repeated measurements of individuals during the study period.
– *Territory ID*: To account for repeated measurements of the same territory across years.

Each model included the following two fixed effects related to vegetation productivity:

– *Territory‐level relative‐NDVI* (described above).
– *Population‐level NDVI* (described above).

To account for other factors likely to influence adult mortality, each model also contained as fixed effects:

– *Year*: Fitted as a continuous covariate ranging from 1994 to 2020.
– *Age*: Restricted to adult birds (1 year old and older). Age was fitted as a quadratic function to account for effects of ageing and senescence on individual mortality. Out of the total 1522 individuals considered in this study, seven individuals (1 female and 6 males) survived to reach age 10 or older. For these rare cases, individuals aged 9 or more were grouped into a single category and treated as age 9+ (following, e.g., Lv et al. 2023).
– *Sex and rank*: Fitted as a three‐level factor: Female, dominant male, and helper male (following, e.g., Lv et al. 2023).
– *Group size*: Representing the maximum number of adult birds alive on a territory during each season (fitted as a covariate ranging from 1 to 7 individuals)
– *Territory size*: Measured in hectares and fitted as a covariate (described above; see also Backhouse et al. 2023).

Note that while we included these fixed effects to control for their influence, our primary focus in this analysis was to assess the effects of spatial and temporal variation in vegetation productivity on adult mortality. Therefore, for completeness, we provide a report of their effects in the results section, but we refrain from extensively interpreting these effects in our discussion.

#### 2.6.4 Female Breeding Success Analyses

The distribution of female breeding success was zero‐inflated (Lv et al. 2019). We therefore constructed a Bayesian hurdle hierarchical generalised linear regression model to assess relationships between vegetation productivity and female breeding success in a given breeding season. The hurdle model comprised two components:

– *Binomial component*: Modelled the probability of a female superb fairy‐wren producing no independent offspring in a given breeding season (i.e., breeding success is 0). Fit with a binomial error distribution and logit‐link function.
– *Conditional component*: Modelled female breeding success for values greater than zero. Fit with a Poisson error distribution and log‐link function.

The hurdle model included the same three random effects as in our adult mortality analyses, plus the fixed effects of territory‐level relative‐NDVI, population‐level NDVI, territory size, and year. Additionally, the following two fixed effects were included, as both have previously been shown to strongly affect different aspects of breeding performance in superb fairy‐wrens (e.g., Cockburn, Sims, et al. 2008; Hajduk et al. 2020, 2021):

– *Female age*: Fitted as a two‐level factor: 1 year old, and 2+ year old (following, e.g., Kruuk et al. 2015; Hajduk et al. 2018).
– *Number of helpers*: Fitted as a three‐level factor: 0 helpers, 1 helper, and 2+ helpers (following, e.g., Cooper et al. 2020; Hajduk et al. 2021; Lv et al. 2019).

Similar to our approach in the adult mortality analysis, our emphasis here was to interpret the effects of vegetation productivity on female breeding success. Therefore, we limit our discussion to exploring these specific effects. It is important to note that for this study, female breeding success was estimated only from territories where the number of helpers remained constant throughout the breeding season (90.96% of the available data). However, when repeating the analyses using the full dataset, excluding the number of helpers as a fixed effect, effectively identical results for the other explanatory parameters were obtained (which we do not present here).

Because superb fairy‐wrens typically forage in understory vegetation (Rowley 1965; Rowley and Russell 1997), we initially included a fixed effect of mean vegetation height in both mortality and breeding success analyses to account for the potential influence of the tree canopy on our NDVI measurements. For completeness, we describe the methods used to calculate mean vegetation height in the Appendix S1: Methods and present these results separately in Tables S5 and S6. However, we excluded this parameter from our main analyses for three reasons: (i) vegetation structure data were available for only 1 year, (ii) some territories lacked vegetation structure data, and (iii) inference of population‐level NDVI and territory‐level relative‐NDVI estimates remained consistent regardless of the inclusion of mean vegetation height in the models.

To account for potential spatial autocorrelation within the data (Dormann et al. 2007), we incorporated a spatial conditional autoregressive (CAR) structure in both the mortality and breeding success analyses. This approach was motivated by previous research conducted on the study population, which showed that locations closer to each other in the study area have more similar structural vegetation characteristics (Turner et al. 2023). The CAR structure was represented as a binary spatial weights matrix (*W*); whereby, the element *W*_*ij*_ of the matrix was set to 1 if the boundaries of territory *i* fell within or overlapped with those of territory *j* (otherwise, 0). To account for potential inaccuracies resulting from hand‐drawn territory boundaries, we applied a 5 m buffer to all territory boundaries before constructing the spatial weights matrix.

In both of the superb fairy‐wren life history analyses, models were fit on 4 independent MCMC chains for 15,000 iterations, with a thinning interval of 24 and a warm‐up period of 3000 iterations (resulting in 2000 posterior samples), specifying weakly informative priors with a normal error distribution (*μ*: 0; *σ* ^2^: 1; Gelman et al. 2015). In all analyses, we visually confirmed model convergence by inspecting the trace plots of parameter estimates and ensuring that potential scale reduction factors were < 1.05 (Gelman et al. 2013; Vehtari et al. 2021). We assessed the fit of each model using the posterior predictive check, *pp_check*, function in the package ‘bayesplot’ (v.1.8.1; Gabry and Mahr 2021). Model parameter estimates are presented as posterior means (±SD) and 95% credible intervals. We considered explanatory parameters to have statistical support when the 95% credible intervals did not overlap zero.

## 3 Results

### 3.1 Spatiotemporal Variation in NDVI

**FIGURE 2:**
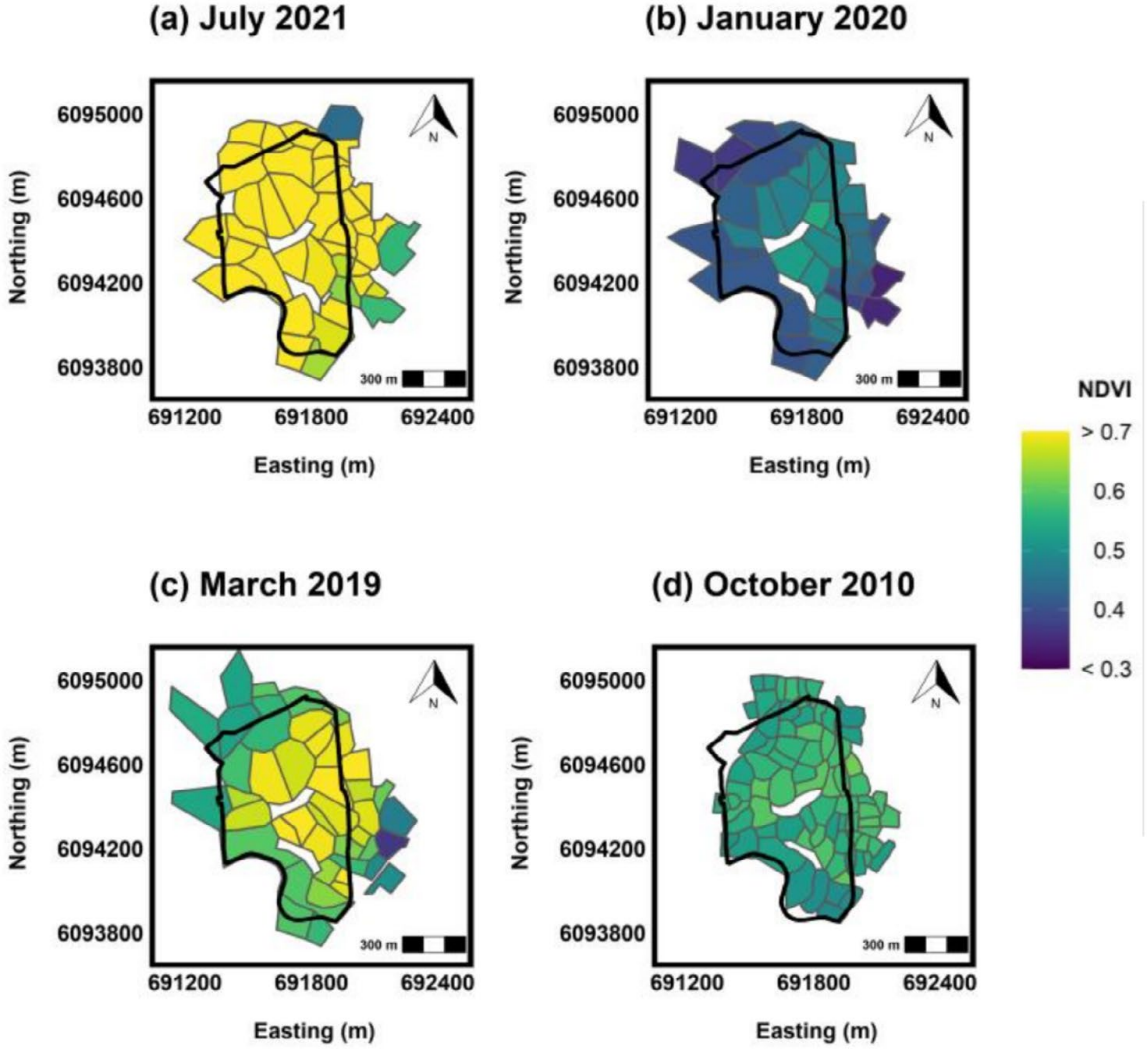
Examples of the spatial and temporal variation in NDVI across the extent of the study area during the study period (1994 to 2020). The panels show the months with the: (a) highest population‐level NDVI (0.73); (b) lowest population‐level NDVI (0.44); (c) highest variation in territory‐level NDVI (SD = 0.08); and (d) lowest variation in territory‐level NDVI (SD = 0.03). In all panels each polygon represents a superb fairy‐wren breeding territory. The perimeter of the Australian National Botanic Gardens (ANBG) is shown in black. Coordinates are projected in metres (m) in spatial reference Geocentric Datum of Australia 1994, Map Grid of Australia Zone 55 (Collier and Steed 2001).

Throughout the study period, there was considerable spatial and temporal variation in both territory‐level and population‐level NDVI values. This variation was observed at monthly, seasonal (i.e., spring–summer and autumn–winter), and annual resolutions (Figures 1, 2, S2; Table S7). The highest monthly population‐level NDVI was in July 2021 (Figure 2a), and the lowest monthly population‐level NDVI was in January 2020 (Figure 2b). Spatial variation in territory‐level relative‐NDVI was least pronounced in March 2019 (Figure 2c), while October 2010 exhibited the greatest spatial variation (Figure 2d). On average, seasonal values of territory‐level and population‐level NDVI were higher during autumn–winter (Figure 1b; Table S7). At an annual resolution, 1997 and 2006 had the lowest population‐level NDVI, while 2020 had the highest population‐level NDVI (Figure S2). Six out of the 7 years with the lowest annual population‐level NDVI values were during prolonged periods of drought in the study area (Figure S2).

### 3.2 Temporal Trends in Weather Conditions and Population‐Level NDVI

**FIGURE 3:**
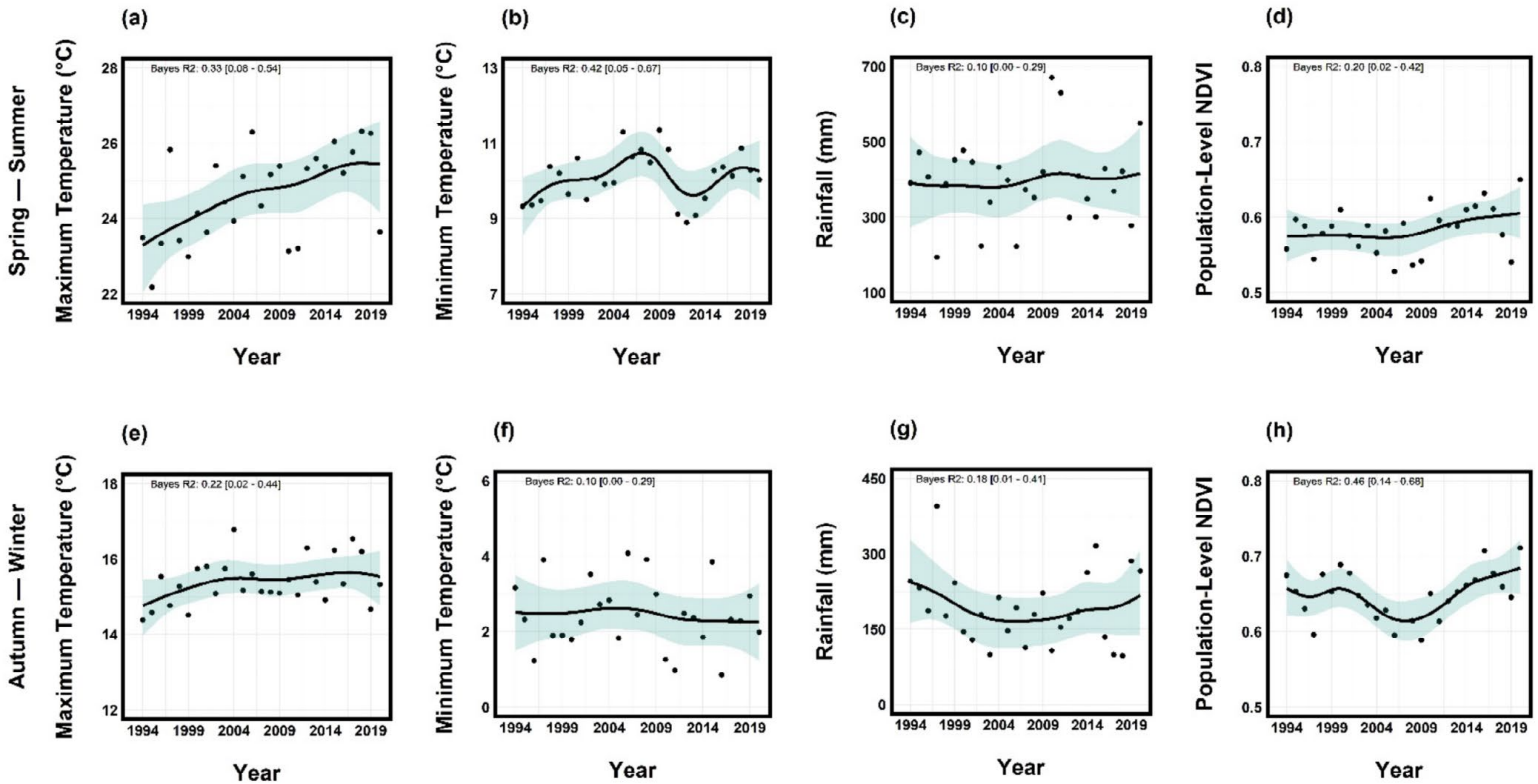
Seasonal changes in (a, e) maximum temperature (°C); (b, f) minimum temperature (°C); (c, g) rainfall; and (d, h) population‐level NDVI during the study period. Regression lines show the model estimated marginal means (±95% confidence intervals). Points show the raw mean for each year. Model estimates are provided in Table S8.

No directional changes were found in minimum temperature, rainfall, or population‐level NDVI across the spring–summer or autumn–winter periods during the study (Figure 3; Table S8). Maximum temperature was found to have increased over time during spring–summer (Figure 3a; Table S8). Although there was no statistical significance in the change of maximum temperature for the autumn–winter period, this was likely due to cooler conditions in the two most recent years (Figure 3e; Table S8). When we excluded data from 2019 and 2020, we did find support for an increase in maximum temperatures in autumn–winter (Estimate ± SD: 0.144 ± 0.054 [95% CI: 0.037 ± 0.248]), consistent with Lv et al. (2023).

### 3.3 Effects of Weather Conditions on NDVI

**FIGURE 4:**
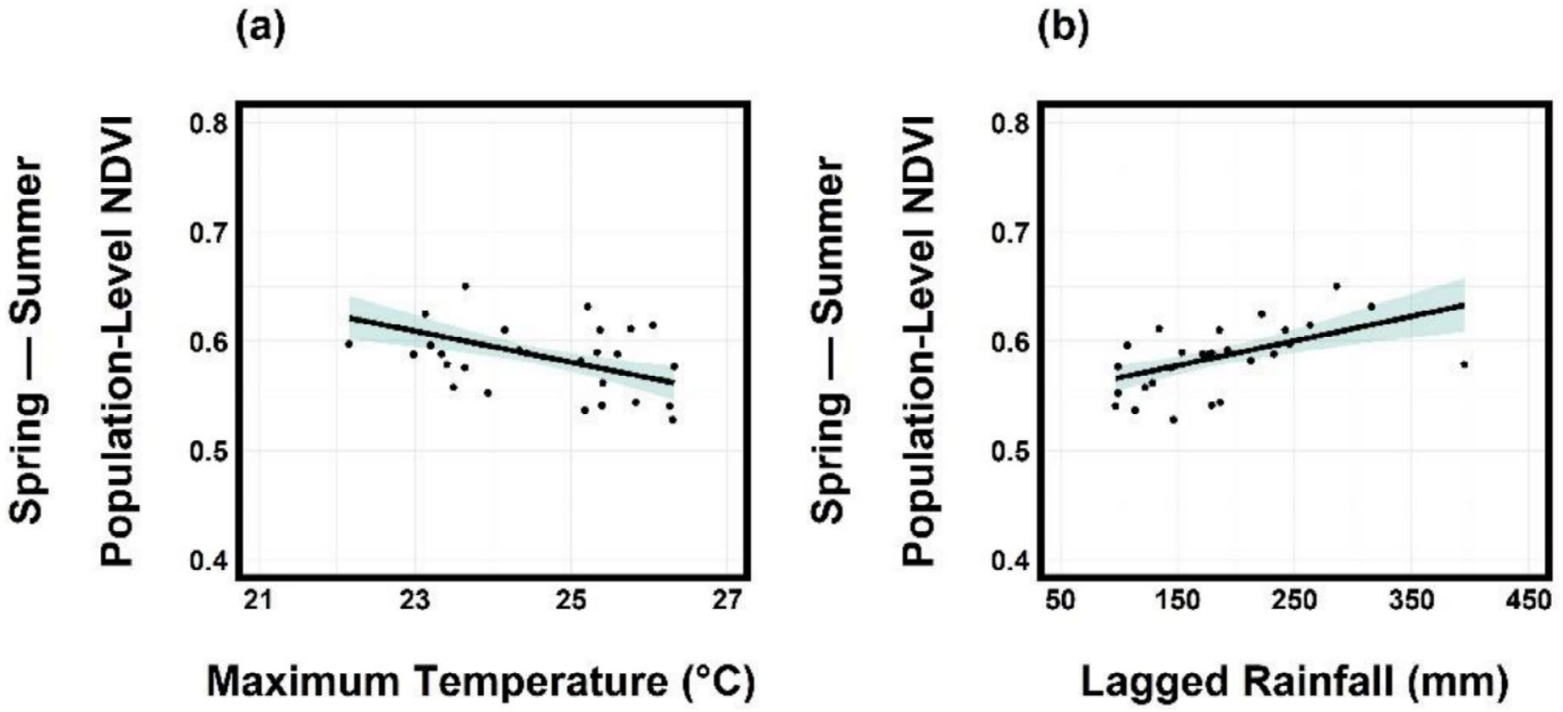
Associations between weather conditions and population‐level NDVI during spring–summer. Regression lines show the model estimated marginal means (±95% confidence intervals) from the top‐ranking model. Model estimates are provided in Table S9.

We observed mixed effects of weather on population‐level NDVI (Tables S4 and S9). Specifically, during spring–summer, the top‐ranking model indicated that population‐level NDVI was positively associated with rainfall in the preceding season, and negatively associated with maximum temperature in the current season (Figure 4; Tables S4 and S9). Although the top‐ranking spring–summer model also included an interaction term between these two weather parameters, the interaction itself was non‐significant (Table S9). Furthermore, removing the interaction term did not significantly impact the model's performance (Table S4). In contrast, when we analysed population‐level NDVI during autumn–winter, we found that the top‐ranking model was a false positive. This was evident from the fact that its ELPD_LOO_ value fell outside the distribution of ELPD_LOO_ values obtained from 100 models fitted with randomised data (i.e., p(ELPD_LOO_) > 0.01; Table S4; Figure S1). Additionally, we found no evidence to suggest that this model had a better fit compared to the baseline model, which had no weather parameters (Table S4).

### 3.4 Adult Mortality

#### 3.4.1 Trends in Adult Mortality Over Time

In our analyses of 1522 adult superb fairy‐wrens (female: *n* = 685; male: *n* = 837), we found that, of all individuals considered across the study period, 95.47% had died by the end of the study, of which 45.25% had died in the spring–summer breeding season period, and 50.20% had died in the autumn–winter non‐breeding season period. The remaining 4.53% of individuals were still alive at the end of the study period. We found no statistical support for any changes over time in adult mortality during either the breeding season or the non‐breeding season (Table 1). For our non‐breeding season analyses, this finding is in contrast to a previous study on our study population (Lv et al. 2023) and is due to exceptionally low rates of adult mortality in the two most recent years (2019 and 2020), which were not considered in Lv et al. (2023) but were included in our analyses.

**TABLE 1:** Summaries of the Bayesian spatial hierarchical generalised linear regression models for adult mortality during the spring–summer (September–March, the superb fairy‐wren breeding season) and autumn–winter (April–August, the superb fairy‐wren non‐breeding season) periods.

Parameters	Spring–summer adult mortality	Autumn–winter adult mortality
Fixed effects	Estimate ± SD (95% CI)	Estimate ± SD (95% CI)
Intercept	**−2.326 ± 0.171 (−2.708, −2.036)**	**−1.538 ± 0.179 (−1.893, −1.206)**
Year	0.021 ± 0.081 (−0.144, 0.184)	0.178 ± 0.158 (−0.126, 0.504)
Group size	**0.233 ± 0.059 (0.124, 0.356)**	−0.067 ± 0.062 (−0.191, 0.056)
Age	**0.696 ± 0.200 (0.402, 1.154)**	**0.639 ± 0.265 (0.171, 1.185)**
Age^2^	**−0.094 ± 0.039 (−0.174, −0.023)**	0.019 ± 0.041 (−0.067, 0.096)
Sex/rank (relative to dominant male)		
Female	**0.902 ± 0.168 (0.611, 1.267)**	−0.022 ± 0.146 (−0.304, 0.274)
Helper male	0.203 ± 0.173 (−0.123, 0.537)	0.149 ± 0.182 (−0.211, 0.508)
Territory‐level relative‐NDVI	−0.076 ± 0.058 (−0.192, 0.040)	**−0.148 ± 0.069 (−0.291, −0.020)**
Population‐level NDVI	0.037 ± 0.070 (−0.098, 0.173)	0.110 ± 0.154 (−0.218, 0.418)
Territory size	−0.018 ± 0.060 (−0.141, 0.097)	**−0.136 ± 0.069 (−0.270, −0.002)**

Random effects	$\sqrt{\text{Variance}}$ ± SD (95% CI)	$\sqrt{\text{Variance}}$ ± SD (95% CI)
Year	0.210 ± 0.087 (0.042, 0.387)	0.707 ± 0.159 (0.455, 1.077)
	(*n* = 27)	(*n* = 26)
Bird ID	0.786 ± 0.410 (0.070, 1.617)	1.303 ± 0.493 (0.231, 2.229)
	(*n* = 1521)	(*n* = 1368)
Territory ID	0.298 ± 0.136 (0.033, 0.554)	0.299 ± 0.157 (0.025, 0.626)
	(*n* = 282)	(*n* = 275)
Spatial correlation	0.407 ± 0.300 (0.016, 1.086)	0.459 ± 0.314 (0.022, 1.176)
	*n* = 4199 samples	*n* = 3449 samples

*Note:* The parameter estimates are presented as posterior means ± standard deviation (SD) and 95% credible intervals (CI). All explanatory parameters were mean standardised for analysis. Fixed effect parameters for which the 95% CI do not overlap zero are highlighted in bold. Number of levels is given below each random effect, and all models were fitted with logit‐link function and parameter estimates are on the link scale. Note: NDVI data were not available during the autumn–winter period of 2007 (which spans March 2008 to August 2008) so this analysis is based on 26 rather than 27 years of data. The effects of territory‐level relative‐NDVI on adult mortality are shown in Figure 5.

#### 3.4.2 Spatial and Temporal Effects of NDVI on Adult Mortality

**FIGURE 5:**
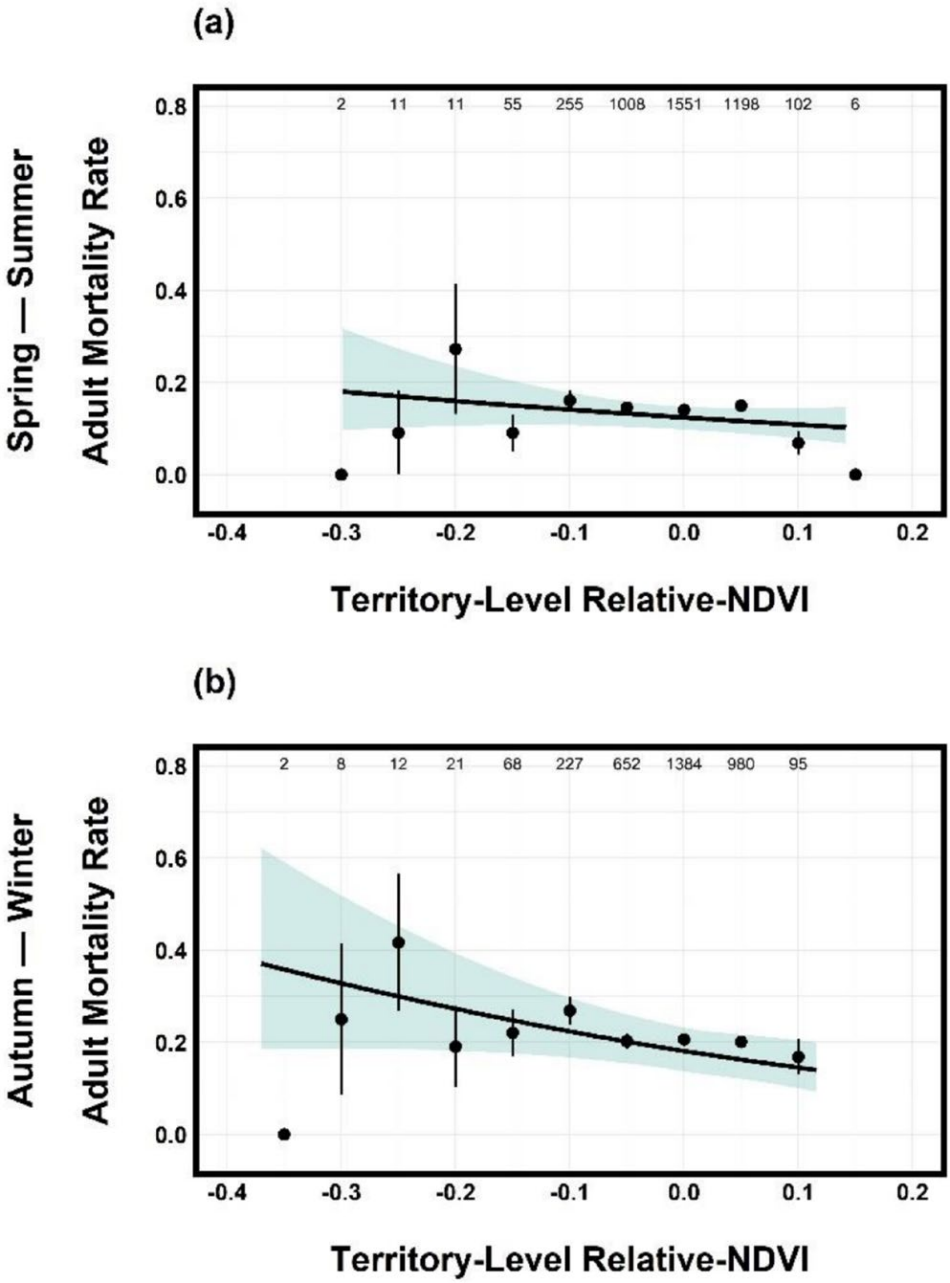
Adult mortality in relation to territory‐level relative NDVI during (a) spring–summer (September–March; the superb fairy‐wren breeding season); and (b) autumn–winter (April–August; the superb fairy‐wren non‐breeding season). Regression lines show the model estimated marginal means (±95% confidence intervals), after correcting for fixed effects, as described in Materials and Methods. For visualisation purposes, the raw data were grouped to the nearest ±0.05 NDVI values with points showing the group mean (±SE). The number of observations in each group is given along the top. Model estimates are provided in Table 1.

We found no evidence to suggest that temporal (i.e., population‐level) variation in NDVI was associated with adult mortality, either during the spring–summer breeding season or the autumn–winter non‐breeding season (Table 1). Similarly, we found no spatial (i.e., territory‐level) effects of NDVI on adult mortality (Figure 5a; Table 1). However, during the non‐breeding season, we found that increased territory‐level relative‐NDVI was associated with lower adult mortality rates (Figure 5b; Table 1).

#### 3.4.3 Effects of Other Social and Demographic Parameters on Adult Mortality

During the spring–summer breeding season, female superb fairy‐wrens had a higher likelihood of dying compared to males, but there was no difference in mortality between males of different rank (Table 1). In contrast, mortality during the autumn–winter non‐breeding season showed no variation based on sex or rank (Table 1). These findings are similar to our monthly resolution adult mortality analyses (Figure 1c; Table S1) and are consistent with a previous study of autumn–winter adult mortality rates in the study population conducted by Lv et al. (2023) (see also Cockburn, Osmond, and Double 2008). Furthermore, adult mortality rates increased linearly with age during the non‐breeding season (Table 1), whereas in the breeding season, adult mortality rates followed a non‐linear pattern, with little change among younger birds but a substantial increase among older birds (Table 1). In terms of social dynamics, larger group size was associated with higher rates of adult mortality in the breeding season, but not during the non‐breeding season (Table 1). Additionally, larger territory size was associated with lower rates of adult mortality, although this effect was only statistically significant during the non‐breeding season (Table 1).

### 3.5 Female Annual Breeding Success

#### 3.5.1 Trends in Female Breeding Success Over Time

There was no evidence of a directional change in female breeding success over time (Table 2).

**TABLE 2:** Summary of the hurdle model of female breeding success (BS; measured by the number of offspring to reach independence in a given breeding season).

Parameters	Probability BS is 0	BS when > 0
Fixed effects	Estimate ± SD (95% CI)	Estimate ± SD (95% CI)
Intercept	−0.026 ± 0.140 (−0.290, 0.243)	**0.573 ± 0.070 (0.431, 0.702)**
Year	0.022 ± 0.111 (−0.193, 0.240)	−0.014 ± 0.049 (−0.116, 0.085)
Territory‐level relative‐NDVI	0.114 ± 0.072 (−0.028, 0.250)	0.014 ± 0.028 (−0.042, 0.066)
Population‐level NDVI	**−0.385 ± 0.105 (−0.599, −0.176)**	**0.151 ± 0.048 (0.057, 0.247)**
Territory size	−0.117 ± 0.078 (−0.272, 0.035)	0.017 ± 0.029 (−0.040, 0.072)
Female age (relative to 1 Year Old)		
2+ years old	**−0.628 ± 0.133 (−0.884, −0.358)**	**0.256 ± 0.062 (0.135, 0.378)**
Number of Helpers (Relative to 0 Helpers)		
1 Helper	−0.305 ± 0.159 (−0.612, 0.005)	**0.182 ± 0.059 (0.068, 0.294)**
2+ Helpers	**−0.880 ± 0.219 (−1.319, −0.441)**	0.121 ± 0.071 (−0.027, 0.256)

Random effects	$\sqrt{\text{Variance}}$ ± SD (95% CI)	$\sqrt{\text{Variance}}$ ± SD (95% CI)
Year	0.373 ± 0.109 (0.178, 0.613)	0.183 ± 0.043 (0.108, 0.275)
	(*n* = 27)	
Female ID	0.314 ± 0.192 (0.019, 0.711)	0.065 ± 0.044 (0.003, 0.162)
	(*n* = 643)	
Territory ID	0.210 ± 0.135 (0.011, 0.492)	0.058 ± 0.042 (0.002, 0.154)
	(*n* = 248)	
Spatial correlation	0.563 ± 0.348 (0.024, 1.262)	0.237 ± 0.091 (0.055, 0.423)
	*n* = 1308 samples	

*Note:* The hurdle model simultaneously estimated the probability of a female producing no independent offspring in a given breeding season (probability BS is 0: fit with a binomial error distribution and logit‐link function) and the number of independent offspring a female produced in a given breeding season, conditional on at least one (BS when > 0: fit with a Poisson —error distribution and a log‐link function). The parameter estimates are presented as posterior means ± standard deviation (SD) and 95% credible intervals (CI). All explanatory parameters were mean standardised for analysis. Fixed effect parameters for which the 95% CI do not overlap zero are highlighted in bold. Note that the parameter estimates for the two parts of the model are not comparable due to different link functions. The effect of population‐level NDVI on female BS is shown in Figure 6.

#### 3.5.2 Spatial and Temporal Effects of NDVI on Female Breeding Success

**FIGURE 6:**
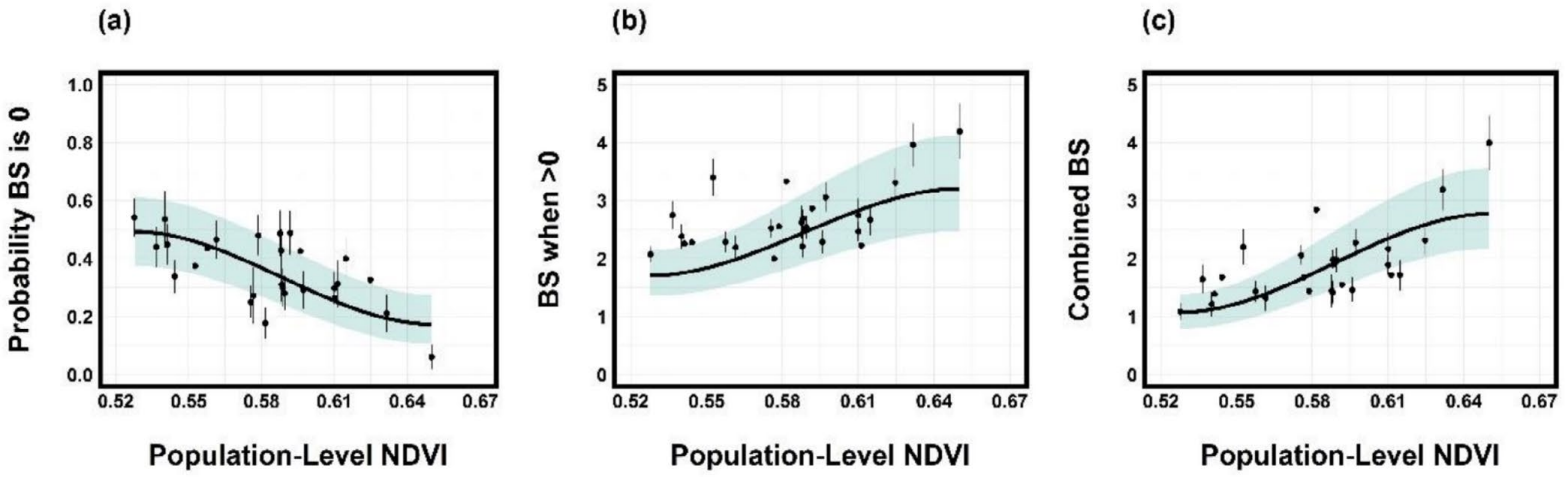
Female breeding success (BS; measured by the number of offspring to reach independence in a given breeding season) in relation to population‐level NDVI. Panel (a) shows the probability a female produced no independent offspring in a given breeding season (i.e., BS is 0; the binomial component of the hurdle model). Panel (b) shows the number of independent offspring a female produced in a given breeding season, when greater than zero (i.e., BS when > 0; the conditional component of the hurdle model). Panel (c) shows the number of independent offspring a female produced in a given breeding season when both components of the hurdle model are combined. In all panels, the regression lines represent the estimated marginal mean (±95% confidence intervals), after correcting for main effect parameters, as described in Materials and Methods. The points show the raw mean (± SE) for each of the 27 years. Model estimates are provided in Table 2.

In terms of spatial variation in NDVI, our analysis did not reveal any evidence of territory‐level relative‐NDVI affecting female breeding success (Table 2). However, there was evidence of temporal variation in effects of NDVI on female breeding success, with increased population‐level NDVI associated with a reduced probability of a female producing no independent offspring in a given breeding season (Table 2; Figure 6a). Furthermore, population‐level NDVI was also positively associated with an increase in the number of independent offspring produced by a female in a given breeding season, conditional on at least one offspring (Table 2; Figure 6b).

#### 3.5.3 Effects of Other Social and Demographic Parameters on Female Breeding Success

Older females and territories with a higher number of helpers were both associated with higher female breeding success (Table 2). Consistent with a previous study of our study population, we found no effect of territory size on female breeding success (Table 2; Backhouse et al. 2023).

## 4 Discussion

We present here an analysis of associations between climate, vegetation, and mortality and breeding success traits in a wild insectivorous passerine bird, the superb fairy‐wren. Our study combined nearly three decades of individual‐level monitoring of superb fairy‐wrens in southeastern Australia with local weather records and Landsat satellite imagery data, from which we derived spatial and temporal measures of NDVI—an index of vegetation productivity (Pettorelli 2013; Rouse et al. 1974) as a proxy for food availability through arthropod abundance (Cole et al. 2015; Fernández‐Tizón et al. 2020). Our results show how effects of climate on vegetation productivity may vary between different seasons, specifically spring–summer and autumn–winter. Through detailed climate analyses, we found a complex set of associations between NDVI and different components of weather, when considering both concurrent and lagged effects. However, we found no evidence of a temporal change in NDVI in the study area, and only limited evidence of climate change across the 27‐year study period (1994 to 2020), with an observed increase in maximum temperatures in spring–summer. Our analyses of superb fairy‐wren life history traits revealed associations between NDVI and spatial variation in adult mortality during autumn–winter (i.e., the superb fairy‐wren non‐breeding season), as well as temporal variation in female breeding success during spring–summer (i.e., the superb fairy‐wren breeding season). Seasonal variation in arthropod abundance identified in other studies (Bell 1985; Fernández‐Tizón et al. 2020) may contribute to these patterns, although other factors may also influence these relationships. We discuss these results in turn below.

### 4.1 Temporal Trends in Weather Conditions and NDVI

During the study period, we observed a significant trend towards higher maximum temperatures in spring–summer that can be attributed to more frequent and intense summer heatwaves (Kruuk et al. 2015; Lv et al. 2019, 2023). However, we did not find statistical support for the observed increase in autumn–winter maximum temperatures reported in Lv et al. (2023). This disparity was due to the relatively milder conditions in 2019 and 2020 (Figure 3e,f), which were not included in previous analyses. Additionally, our findings revealed no significant directional change in minimum temperature or rainfall for both spring–summer and autumn–winter, consistent with Lv et al. (2019, 2023). The absence of a linear rainfall trend can be attributed to two periods of drought recorded in the study area: one between 2001 and 2009 (Murphy and Timbal 2008; van Dijk et al. 2013) and another more recently from 2017 to 2019. In line with these observed variable weather trends, we found no evidence of any temporal linear change in NDVI.

### 4.2 Seasonal Effects of Climate on Vegetation Productivity

#### 4.2.1 Spring–Summer

We found a positive association between rainfall during the previous autumn–winter and NDVI in the subsequent spring–summer, indicative of a long‐term beneficial impact of rainfall on vegetation productivity in the study area. Conversely, we found that higher spring–summer maximum temperatures had a negative effect on spring–summer NDVI. Despite the observed increase in maximum temperatures during spring–summer throughout the study period, there was no directional change in spring–summer NDVI. These findings therefore suggest that existing rainfall patterns during autumn–winter may mitigate the adverse effects associated with climate warming in spring–summer.

#### 4.2.2 Autumn–Winter

We did not find robust evidence of associations between NDVI and autumn–winter climate. During these analyses, we identified a false positive in the top‐ranking model. Furthermore, this model failed to outperform the baseline model, which did not consider weather conditions. These findings suggest that maximum temperature, minimum temperature, and rainfall in the current and previous season do not have an impact on vegetation productivity during autumn–winter.

Interestingly, we observed substantial variation in NDVI throughout the year and between years. Notably, there was a trend towards higher NDVI values in autumn–winter compared to spring–summer (Table S7). However, these trends were not quantitatively assessed and may have been influenced by cloud cover. The majority of unusable Landsat imagery acquired for this study was from the autumn–winter months. Out of the 33 months' worth of unusable autumn–winter images, 23 months (69.70%) were between June and August, which corresponds to a period of increased autumn–winter rainfall (Figure 1a), and hence likely cloudier conditions.

### 4.3 Determinants of Adult Mortality

In our analyses of adult mortality rates during the spring–summer breeding season and the autumn–winter non‐breeding season, we found no evidence of significant associations between population‐level NDVI and mortality. Initially, this finding suggests that changes in arthropod abundance do not impact the mortality rates of adult superb fairy‐wrens.

However, when we considered spatial variation in NDVI, we found a negative association between territory‐level relative‐NDVI and autumn–winter adult mortality (Figure 5b). This finding implies that individual superb fairy‐wrens face a higher risk of mortality in a given non‐breeding season if they inhabit territories with on average relatively lower vegetation productivity, and thus presumably relatively lower food abundance. Interestingly, the significance of territory‐level relative‐NDVI was only observed during autumn–winter. This finding is significant because in temperate regions, the autumn–winter period corresponds to the time of year with the lowest arthropod biomass (Bell 1985; Fernández‐Tizón et al. 2020). Additionally, it is a period when thermoregulation is most important to combat cold weather (Swanson 2001; Swanson and Olmstead 1999; Zhao et al. 2015; Zheng, Li, et al. 2008, Zheng, Liu, et al. 2008). Our results therefore suggest that, for superb fairy‐wrens inhabiting less productive territories, there is a higher likelihood of death due to starvation or cold stress if individuals are unable to replenish their energy reserves during this time when food is scarce. Moreover, individuals in such territories may need to allocate more time to foraging, which can limit their ability to effectively monitor and defend against predators, thereby increasing the risk of predation (Caraco 1979; Lima 1988).

### 4.4 Female Breeding Success

In years with higher spring–summer population‐level NDVI values, female superb fairy‐wrens had higher breeding success (Figure 6a–c). Previous research conducted on the study population has shown that warmer and drier conditions during spring and summer lead to a decline in nestling body mass (Kruuk et al. 2015). Smaller body mass has a negative impact on the survival of the nestlings in this population after they have fledged (Hajduk et al. 2020) and studies on various other bird species have similarly shown that heavier nestlings are more likely to fledge and survive to breed themselves (Bourne et al. 2020; Magrath 1991; van de Ven et al. 2020). Furthermore, Cockburn, Osmond, and Double (2008) found a positive association between breeding success and spring–summer rainfall. Our findings align with these previous studies and suggest that variation in vegetation productivity over time, and thus arthropod abundance, may explain variation in breeding success.

However, weather conditions may also influence breeding success through other mechanisms. For example, Lv et al. (2019) found that milder and wetter conditions can extend the length of the superb fairy‐wren breeding season and increase the number of breeding attempts. Daily predation rates of superb fairy‐wren nests are also lower during the early and late stages of the breeding season, which could increase breeding success in years when the breeding season starts earlier or is prolonged (Turner et al. 2024). Furthermore, Oswald et al. (2021) found that adult birds of the Cape rockjumper *Chaetops frenatus* reduced their provisioning rates of nestlings on warmer days, resulting in decreased nestling body mass. If this behaviour also occurs in superb fairy‐wrens, changes in nestling provisioning rates could also affect breeding success regardless of any changes in food availability.

Our analysis of spatial variation in NDVI did not provide evidence to support the notion that territory‐level relative‐NDVI had an impact on female breeding success. One possible explanation for this outcome is that habitats with higher vegetation productivity tend to support a greater abundance and diversity of species compared to less productive habitats (McKinney 2008; Wesche et al. 2012). If this pattern holds true across the study area, it is possible that increased interspecific competition for food (and other habitat resources) on territories with higher NDVI values may counteract any benefits of occupying such territories, at least in terms of breeding success (Jones et al. 2014; but see, Dhondt 2010). Future studies are needed to gain a more comprehensive understanding of the factors influencing breeding success in superb fairy‐wrens and the underlying mechanisms driving these relationships.

## 5 Conclusion

The aim of this study was to investigate the utility of freely accessible and long‐term Landsat satellite imagery data for assessing the effects of climate change on vegetation productivity and associated variation in life history traits of superb fairy‐wrens. Our findings revealed complex associations between climate and vegetation productivity, both at different times of the year and between years. We also found that vegetation productivity varied considerably within the study area, and that these spatial and temporal patterns of vegetation productivity influence superb fairy‐wren life history traits during different seasons. Despite the spectral and spatiotemporal limitations of Landsat imagery, we illustrate that these data have great potential when combined with datasets from long‐term animal studies to provide retrospective measures of influential ecological conditions over time that may not have been collected or available otherwise.

## Author Contributions

**Richard S. Turner:** conceptualization (equal), data curation (supporting), formal analysis (lead), methodology (equal), visualization (lead), writing – original draft (lead), writing – review and editing (equal). **Ophélie J. D. Lasne:** data curation (supporting), formal analysis (supporting), methodology (supporting). **Lei Lv:** formal analysis (supporting), methodology (supporting). **Helen L. Osmond:** data curation (equal), methodology (supporting). **Andrew Cockburn:** conceptualization (equal), data curation (equal), funding acquisition (equal), methodology (supporting), writing – review and editing (supporting). **Loeske E. B. Kruuk:** conceptualization (equal), formal analysis (supporting), funding acquisition (equal), methodology (equal), supervision (lead), writing – original draft (supporting), writing – review and editing (supporting). **Kara N. Youngentob:** conceptualization (equal), formal analysis (supporting), methodology (equal), supervision (supporting), writing – original draft (supporting), writing – review and editing (equal).

## Funding

This work was supported by the Australian Research Council, DP190100424.

## Conflicts of Interest

The authors declare no conflicts of interest.

## Supporting information


**Figure S1:** Randomisation tests for top‐ranked models.
**Figure S2:** Variation in NDVI over time.
**Table S1:** Summaries of the Bayesian spatial hierarchical generalised linear regression models for monthly resolution adult mortality during the spring–summer superb fairy‐wren breeding season (September to March), the autumn–winter superb fairy‐wren non‐breeding season (April to August), and across the full year.
**Table S2:** Summary of the Bayesian spatial hierarchical generalised linear regression model for annual resolution adult mortality.
**Table S3:** Selection of baseline Bayesian hierarchical generalised linear regression models to account for non‐weather‐related effects on population‐level NDVI during the spring–summer (September to March) and autumn–winter (April to August).
**Table S4:** Model selection to determine the weather conditions that best predicted population‐level NDVI.
**Table S5:** Summaries of the Bayesian spatial hierarchical generalised linear regression models for adult mortality during the spring–summer (September–March, the superb fairy‐wren breeding season) and autumn–winter (April–August, the superb fairy‐wren non‐breeding season) periods, accounting for the potential influence of the tree canopy (mean vegetation height) on NDVI.
**Table S6:** Summary of the hurdle model of female breeding success (BS; measured by the number of offspring to reach independence in a given breeding season), accounting for the potential influence of the tree canopy (mean vegetation height) on NDVI.
**Table S7:** Spatial and temporal variation in NDVI values during our study period.
**Table S8:** Summaries of the eight Bayesian Generalised Additive Models.
**Table S9:** Summaries of the two top‐ranked Bayesian linear regression models of population‐level NDVI when testing for weather effects.

## Data Availability

Data needed to evaluate the conclusions presented in this study are available at https://doi.org/10.6084/m9.figshare.31353343. Landsat imagery is available from Digital Earth Australia at https://ga.gov.au/scientific‐topics/dea.
