# Supplementary material for: Using Landsat Satellite Imagery to Investigate Spatial and Temporal Variation in Life History Traits in a Long‐Term Study Population of Superb Fairy‐Wrens Malurus cyaneus
Source: Ecol Evol. 2026 Jun 14;16(6):e73852. doi: 10.1002/ece3.73852 (PMC13265397; doi:10.1002/ece3.73852)
Supplement: Supplementary file 1 — Figure S1: Randomisation tests for top‐ranked models. Figure S2: Variation in NDVI over time. Table S1: Summaries of the Bayesian spatial hierarchical generalised linear regression models for monthly resolution adult mortality during the spring–summer superb fairy‐wren breeding season (September to March), the autumn–winter superb fairy‐wren non‐breeding season (April to August), and across the full year. Table S2: Summary of the Bayesian spatial hierarchical generalised linear regression model for annual resolution adult mortality. Table S3: Selection of baseline Bayesian hierarchical generalised linear regression models to account for non‐weather‐related effects on population‐level NDVI during the spring–summer (September to March) and autumn–winter (April to August). Table S4: Model selection to determine the weather conditions that best predicted population‐level NDVI. Table S5: Summaries of the Bayesian spatial hierarchical generalised linear regression models for adult mortality during the spring–summer (September–March, the superb fairy‐wren breeding season) and autumn–winter (April–August, the superb fairy‐wren non‐breeding season) periods, accounting for the potential influence of the tree canopy (mean vegetation height) on NDVI. Table S6: Summary of the hurdle model of female breeding success (BS; measured by the number of offspring to reach independence in a given breeding season), accounting for the potential influence of the tree canopy (mean vegetation height) on NDVI. Table S7: Spatial and temporal variation in NDVI values during our study period. Table S8: Summaries of the eight Bayesian Generalised Additive Models. Table S9: Summaries of the two top‐ranked Bayesian linear regression models of population‐level NDVI when testing for weather effects. [file ECE3-16-e73852-s001.docx]

**SUPPLEMENTARY INFORMATION**

***Table S1:*** *Summaries of the Bayesian spatial hierarchical generalised linear regression models for monthly resolution adult mortality during the spring–summer superb fairy-wren breeding season (September to March), the autumn–winter superb fairy-wren non-breeding season (April to August), and across the full year.*

|  |  |  |  |
| --- | --- | --- | --- |
|  | **Spring–Summer**  **Adult Mortality** | **Autumn–Winter**  **Adult Mortality** | **Annual**  **Adult Mortality** |
| Fixed Effects | Estimate ± SD (95% CI) | Estimate ± SD (95% CI) | Estimate ± SD (95% CI) |
| Intercept | **-4.375 ± 0.190 (-4.773, -4.040)** | **-3.371 ± 0.204 (-3.793, -2.992)** | **-4.152 ± 0.120 (-4.379, -3.923)** |
| Year | 0.041 ± 0.080 (-0.114, 0.195) | 0.113 ± 0.127 (-0.146, 0.361) | 0.062 ± 0.064 (-0.058, 0.192) |
| Month | 0.236 ± 0.218 (-0.168, 0.672) | **0.953 ± 0.217 (0.524, 1.401)** | **0.368 ± 0.185 (0.005, 0.719)** |
| Month^2^ | -0.027 ± 0.105 (-0.232, 0.174) | -0.062 ± 0.123 (-0.295, 0.186) | **0.179 ± 0.083 (0.019, 0.340)** |
| Month^3^ | -0.025 ± 0.115 (-0.255, 0.197) | **-0.263 ± 0.131 (-0.521, -0.009)** | 0.073 ± 0.084 (-0.086, 0.235) |
| Group Size | **0.243 ± 0.055 (0.139, 0.352)** | -0.019 ± 0.055 (-0.127, 0.089) | **0.116 ± 0.040 (0.035, 0.196)** |
| Age | **0.588 ± 0.145 (0.344, 0.902)** | **0.314 ± 0.141 (0.110, 0.660)** | **0.431 ± 0.111 (0.255, 0.669)** |
| Age^2^ | **-0.099 ± 0.038 (-0.176, -0.031)** | 0.012 ± 0.032 (-0.057, 0.073) | -0.038 ± 0.025 (-0.088, 0.010) |
| Rank  (Relative to Dominant Male) |  |  |  |
| *Female* | **1.245 ± 0.182 (0.904, 1.622)** | 0.195 ± 0.175 (-0.144, 0.552) | **0.711 ± 0.130 (-0.456, 0.959)** |
| *Helper Male* | 0.073 ± 0.234 (-0.387, 0.526) | -0.008 ± 0.238 (-0.483, 0.444) | -0.288 ± 0.189 (-0.674, 0.083) |
| Territory-Level Relative-NDVI | -0.035 ± 0.057 (-0.142, 0.077) | -0.094 ± 0.052 (-0.196, 0.010) | -0.069 ± 0.036 (-0.138, 0.004) |
| Population-Level NDVI | -0.038 ± 0.074 (-0.183, 0.109) | -0.055 ± 0.098 (-0.252, 0.130) | 0.034 ± 0.076 (-0.116, 0.176) |
| Territory Size | -0.013 ± 0.060 (-0.134, 0.098) | -0.117 ± 0.063 (-0.240, 0.004) | -0.059 ± 0.042 (-0.141, 0.020) |
| Rank: Month |  |  |  |
| *Female: Month* | **-0.771 ± 0.257 (-1.264, -0.278)** | -0.096 ± 0.274 (-0.605, 0.440) | **-0.747 ± 0.171 (-1.092, -0.414)** |
| *Helper Male: Month* | -0.383 ± 0.345 (-1.085, 0.257) | -0.324 ± 0.378 (-1.107, 0.406) | -0.342 ± 0.241 (-0.804, 0.137) |
| Rank: Month^2^ |  |  |  |
| *Female: Month^2^* | **-0.417 ± 0.126 (-0.663, -0.171)** | -0.160 ± 0.156 (-0.470, -0.146) | -0.163 ± 0.094 (-0.349, 0.024) |
| *Helper Male: Month^2^* | 0.077 ± 0.164 (-0.252, 0.387) | -0.215 ± 0.232 (-0.658, 0.217) | **0.340 ± 0.119 (0.114, 0.582)** |
| Rank: Month^3^ |  |  |  |
| *Female: Month^3^* | **0.402 ± 0.142 (0.129, 0.687)** | 0.065 ± 0.173 (-0.274, 0.416) | **0.228 ± 0.088 (0.061, 0.409)** |
| *Helper Male: Month^3^* | -0.030 ± 0.179 (-0.360, 0.320) | 0.424 ± 0.240 (-0.050, 0.893) | 0.004 ± 0.115 (-0.217, 0.235) |
| Random Effects | $\sqrt{\text{Variance}}$ ± SD (95% CI) | $\sqrt{\text{Variance}}$ ± SD (95% CI) | $\sqrt{\text{Variance}}$ ± SD (95% CI) |
| Year | 0.151 ± 0.095 (0.007, 0.349) | 0.556 ± 0.125 (0.337, 0.820) | 0.171 ± 0.088 (0.013, 0.354) |
|  | (n = 27) | (n = 26) | (n = 27) |
| Month: Year | 0.384 ± 0.083 (0.222, 0.535) | 0.260 ± 0.106 (0.036, 0.456) | 0.502 ± 0.054 (0.400, 0.608) |
|  | (n = 158) | (n = 102) | (n = 260) |
| Bird ID | 0.682 ± 0.307 (0.084, 1.266) | 0.576 ± 0.376 (0.023, 1.356) | 0.445 ± 0.226 (0.031, 0.873) |
|  | (n = 1521) | (n = 1361) | (n = 1522) |
| Territory ID | 0.383 ± 0.114 (0.121, 0.582) | 0.195 ± 0.114 (0.014, 0.427) | 0.193 ± 0.084 (0.024, 0.340) |
|  | (n = 282) | (n = 274) | (n = 282) |
| Spatial Correlation | 0.337 ± 0.270 (0.012, 0.997) | 0.233 ± 0.182 (0.013, 0.690) | 0.228 ± 0.180 (0.008, 0.656) |
|  | n = 22321 samples | n = 12386 samples | n = 34707 samples |

*The parameter estimates are presented as posterior means ± standard deviation (SD) and 95% credible intervals (CI). All explanatory parameters were mean standardised for analysis. Main effect parameters for which the 95% CI do not overlap zero are highlighted in bold. Note: NDVI data were not available for any months during the autumn–winter of 2007 (which spans March 2008–August 2008) so this analysis is based on 26 years of adult mortality data only. Bird ID sample sizes differ between the spring–summer and annual analyses due to one bird migrating into the study area during the winter but dying before the start of the following breeding season.*

***Table S2:*** *Summary of the Bayesian spatial hierarchical generalised linear regression model for annual resolution adult mortality.*

|  |  |
| --- | --- |
| **Parameters** | **Annual Adult Mortality** |
| Fixed Effects | Estimate ± SD (95% CI) |
| Intercept | **-0.791 ± 0.136 (-1.030, -0.512)** |
| Year | 0.114 ± 0.089 (-0.056, 0.300) |
| Group Size | **0.111 ± 0.048 (0.021, 0.209)** |
| Age | **0.701 ± 0.239 (0.332, 1.230)** |
| Age^2^ | -0.031 ± 0.034 (-0.103, 0.031) |
| Rank  (Relative to Dominant Male) |  |
| *Female* | **0.422 ± 0.115 (0.222, 0.655)** |
| *Helper Male* | 0.114 ± 0.132 (-0.139, 0.382) |
| Territory-Level Relative-NDVI | -0.090 ± 0.048 (-0.183, 0.003) |
| Population-Level NDVI | 0.070 ± 0.085 (-0.100, 0.242) |
| Territory Size | -0.088 ± 0.048 (-0.185, 0.006) |
| Random Effects | $\sqrt{\text{Variance}}$ ± SD (95% CI) |
| Year | 0.361 ± 0.084 (0.223, 0.551) |
|  | (n = 27) |
| Bird ID | 0.868 ± 0.350 (0.196, 1.562) |
|  | (n = 1522) |
| Territory ID | 0.203 ± 0.104 (0.015, 0.414) |
|  | (n = 282) |
| Spatial Correlation | 0.294 ± 0.214 (0.013, 0.790) |
|  | n = 4203 samples |

*The parameter estimates are presented as posterior means ± standard deviation (SD) and 95% credible intervals (CI). All explanatory parameters were mean standardised for analysis. Fixed effect parameters for which the 95% CI do not overlap zero are highlighted in bold.*

***Table S3:*** *Selection of baseline Bayesian hierarchical generalised linear regression models to account for non-weather-related effects on population-level NDVI during the spring–summer (September to March) and autumn–winter (April to August).*

| **Spring–Summer** |  |  |  |
| --- | --- | --- | --- |
| **Model Structure** | **ELPD _LOO_ ± SE** | **ΔELPD _LOO_ ± SE** | **LOO _IC_ ± SE** |
| **Year** | **54.505 ± 3.240** |  | **-109.011 ± 6.480** |
| Year + Year^2^ | 53.445 ± 3.970 | -1.060 ± 1.730 | -106.891 ± 7.939 |
| Year + Year^2^ + Year^3^ | 51.769 ± 4.134 | -2.736 ± 1.780 | -103.539 ± 8.267 |
| Year + Year^2^ + Year^3^ + Year^4^ | 50.750 ± 4.993 | -3.755 ± 3.023 | -101.501 ± 9.985 |
| **Autumn–Winter** |  |  |  |
| **Model Structure** | **ELPD _LOO_ ± SE** | **ΔELPD _LOO_ ± SE** | **LOO _IC_ ± SE** |
| Year + Year^2^ | 53.314 ± 3.108 |  | -106.627 ± 6.215 |
| Year + Year^2^+ Year^3^ | 52.365 ± 3.032 | -0.949 ± 0.702 | -104.729 ± 6.065 |
| Year + Year^2^ + Year^3^ + Year^4^ | 51.205 ± 4.104 | -2.109 ± 2.600 | -102.410 ± 8.209 |
| **Year** | **50.448 ± 2.798** | **-2.866 ± 2.464** | **-100.895 ± 5.596** |

*Models were ranked based on their posterior Expected Log Predictive Density (ELPD _LOO_), which is a Bayesian leave-one-out (LOO) measure of expected predictive accuracy. The difference in ELPD _LOO_ between the best fitting model and the model under consideration (ΔELPD _LOO_) was used to assess the significance of model differences. LOO _IC_ is the ELPD _LOO_ on the deviance scale (i.e., -2 x ELPD _LOO_; Vehtari et al., 2017). SE is the computed standard errors for each measurement. In both analyses, the ΔELPD _LOO_ was found to be less than 4 for all models, indicating negligible differences. Therefore, we selected the most parsimonious model for each final baseline model, which is highlighted in bold. Model comparisons were implemented using the loo function in the package ‘loo’ (v.2.4.1; Vehtari et al., 2020) in R (v.4.0.5; R Core Team, 2021).*

***Table S4:*** *Model selection to determine the weather conditions that best predicted population-level NDVI.*

| **Spring–Summer** |  | |  | |  | |  | |
| --- | --- | --- | --- | --- | --- | --- | --- | --- |
| **Model Structure** | **ELPD _LOO_ ± SE** | **ΔELPD _LOO_ ± SE** | | **LOO ± SE** | | **False Positive?** | |  |
| Rainfall (Lagged) + Maximum Temperature + Rainfall (Lagged): Maximum Temperature | 66.413 ± 3.378 |  | | -132.825 ± 6.757 | | N | |  |
| Rainfall (Lagged) + Maximum Temperature | 64.960 ± 4.163 | -1.452 ± 2.163 | | -129.921 ± 8.326 | |  | |  |
| **Baseline** | **54.505 ± 3.240** | **-11.907 ± 3.943** | | **-109.011 ± 6.480** | |  | |  |
| **Autumn–Winter** |  | |  | |  | |  | |
| **Model Structure** | **ELPD _LOO_ ± SE** | **ΔELPD _LOO_ ± SE** | | **LOO ± SE** | | **False Positive?** | |  |
| Rainfall (Lagged) + Maximum Temperature (Lagged) + Rainfall (Lagged): Maximum Temperature (Lagged) | 51.957 ± 3.416 |  | | -103.914 ± 6.831 | | Y | |  |
| Minimum Temperature | 51.679 ± 2.990 | -0.278 ± 2.865 | | -103.358 ± 5.979 | |  | |  |
| Maximum Temperature (Lagged) | 51.642 ± 3.162 | -0.315 ± 2.164 | | -103.283 ± 6.325 | |  | |  |
| Rainfall (Lagged) + Maximum Temperature (Lagged) | 50.852 ± 3.220 | -1.105 ± 1.900 | | -101.703 ± 6.440 | |  | |  |
| Rainfall + Maximum Temperature (Lagged) | 50.748 ± 2.968 | -1.209 ± 2.245 | | -101.497 ± 5.936 | |  | |  |
| Rainfall + Minimum Temperature (Lagged) + Rainfall: Minimum Temperature (Lagged) | 50.631 ± 2.565 | -1.326 ± 3.326 | | -101.262 ± 5.130 | |  | |  |
| Rainfall (Lagged) + Minimum Temperature + Rainfall (Lagged): Minimum Temperature | 50.574 ± 3.194 | -1.383 ± 2.392 | | -101.149 ± 6.388 | |  | |  |
| Rainfall + Maximum Temperature (Lagged) + Rainfall: Maximum Temperature (Lagged) | 50.480 ± 2.726 | -1.477 ± 1.936 | | -100.961 ± 5.452 | |  | |  |
| **Baseline** | **50.448 ± 2.798** | **-1.509 ± 2.746** | | **-100.895 ± 5.596** | |  | |  |
| Rainfall + Minimum Temperature | 50.302 ± 2.919 | -1.654 ± 2.945 | | -100.605 ± 5.837 | |  | |  |
| Rainfall (Lagged) + Minimum Temperature | 50.183 ± 3.095 | -1.774 ± 3.064 | | -100.366 ± 6.189 | |  | |  |
| Rainfall (Lagged) | 50.105 ± 3.064 | -1.852 ± 2.320 | | -100.210 ± 6.129 | |  | |  |
| Minimum Temperature (Lagged) | 50.070 ± 2.475 | -1.887 ± 2.475 | | -100.139 ± 5.423 | |  | |  |
| Rainfall | 49.596 ± 2.755 | -2.361 ± 2.975 | | -99.192 ± 5.511 | |  | |  |
| Rainfall + Minimum Temperature + Rainfall: Minimum Temperature | 49.466 ± 2.759 | -2.491 ± 2.806 | | -98.932 ± 5.518 | |  | |  |
| Rainfall + Minimum Temperature (Lagged) | 49.430 ± 2.743 | -2.526 ± 2.810 | | -98.861 ± 5.486 | |  | |  |
| Maximum Temperature | 49.415 ± 2.712 | -2.541 ± 2.691 | | -98.831 ± 5.424 | |  | |  |
| Rainfall (Lagged) + Maximum Temperature | 49.122 ± 2.685 | -2.835 ± 2.228 | | -98.244 ± 5.729 | |  | |  |
| Rainfall (Lagged) + Minimum Temperature (Lagged) | 49.068 ± 3.695 | -2.889 ± 2.875 | | -98.136 ± 7.391 | |  | |  |
| Rainfall + Maximum Temperature | 48.586 ± 2.676 | -3.371 ± 2.812 | | -97.172 ± 5.352 | |  | |  |
| Rainfall (Lagged) + Minimum Temperature (Lagged) + Rainfall (Lagged): Minimum Temperature | 48.030 ± 3.445 | -3.927 ± 2.978 | | -96.060 ± 6.891 | |  | |  |

*Lagged demonstrates that weather data were from the preceding season. Models were ranked based on their posterior Expected Log Predictive Density (ELPD _LOO_). The difference in ELPD _LOO_ between the best fitting model and the model under consideration (ΔELPD _LOO_) was used to assess the significance of model differences. LOO _IC_ is the ELPD _LOO_ on the deviance scale (i.e., -2 x ELPD _LOO_; Vehtari et al., 2017). SE is the computed standard errors for each measurement. The baseline model for each analysis is highlighted in bold. Model comparisons were implemented using the loo function in the package ‘loo’ (v.2.4.1; Vehtari et al., 2020) in R (v.4.0.5; R Core Team, 2021).*

***Table S5:*** *Summaries of the Bayesian spatial hierarchical generalised linear regression models for adult mortality during the spring–summer (September–March, the superb fairy-wren breeding season) and autumn–winter (April–August, the superb fairy-wren non-breeding season) periods, accounting for the potential influence of the tree canopy (mean vegetation height) on NDVI.*

|  |  |  |
| --- | --- | --- |
| **Parameters** | **Spring–Summer Adult Mortality** | **Autumn–Winter Adult Mortality** |
| Fixed Effects | Estimate ± SD (95% CI) | Estimate ± SD (95% CI) |
| Intercept | **-2.273 ± 0.162 (-2.622, -1.991)** | **-1.555 ± 0.169 (-1.908, -1.240)** |
| Year | 0.041 ± 0.083 (-0.128, 0.202) | 0.164 ± 0.150 (-0.126, 0.469) |
| Group Size | **0.226 ± 0.062 (0.112, 0.355)** | -0.083 ± 0.061 (-0.200, 0.040) |
| Age | **0.660 ± 0.172 (0.407, 1.046)** | **0.423 ± 0.221 (0.105, 0.941)** |
| Age^2^ | **-0.104 ± 0.040 (-0.187, -0.029)** | 0.049 ± 0.039 (-0.027, 0.124) |
| Sex/Rank  (Relative to Dominant Male) |  |  |
| *Female* | **0.896 ± 0.157 (0.607, 1.226)** | -0.094 ± 0.136 (-0.370, 0.170) |
| *Helper Male* | 0.147 ± 0.175 (-0.210, 0.481) | 0.135 ± 0.173 (-0.204, 0.492) |
| Territory-Level Relative-NDVI | -0.056 ± 0.069 (-0.191, 0.078) | **-0.202 ± 0.075 (-0.357, -0.064)** |
| Population-Level NDVI | 0.026 ± 0.073 (-0.125, 0.168) | 0.124 ± 0.145 (-0.168, 0.409) |
| Territory Size | -0.002 ± 0.061 (-0.125, 0.115) | **-0.137 ± 0.067 (-0.268, -0.007)** |
| Mean Vegetation Height | -0.033 ± 0.070 (-0.168, 0.107) | 0.111 ± 0.078 (-0.036, 0.273) |
| Random Effects | $\sqrt{\text{Variance}}$ ± SD (95% CI) | $\sqrt{\text{Variance}}$ ± SD (95% CI) |
| Year | 0.220 ± 0.094 (0.035, 0.410) | 0.654 ± 0.145 (0.413, 0.971) |
|  | (n = 27) | (n = 26) |
| Bird ID | 0.671 ± 0.377 (0.051, 1.437) | 0.882 ± 0.483 (0.066, 1.854) |
|  | (n = 1388) | (n = 1251) |
| Territory ID | 0.324 ± 0.139 (0.036, 0.588) | 0.268 ± 0.144 (0.016, 0.557) |
|  | (n = 257) | (n = 251) |
| Spatial Correlation | 0.477 ± 0.338 (0.023, 1.230) | 0.481 ± 0.301 (0.031, 1.140) |
|  | n = 3812 samples | n = 3131 samples |

***Table S6:*** *Summary of the hurdle model of female breeding success (BS; measured by the number of offspring to reach independence in a given breeding season), accounting for the potential influence of the tree canopy (mean vegetation height) on NDVI.*

|  |  |  |
| --- | --- | --- |
| **Parameters** | **Probability BS is 0** | **BS when >0** |
| Fixed Effects | Estimate ± SD (95% CI) | Estimate ± SD (95% CI) |
| Intercept | -0.037 ± 0.141 (-0.329, 0.230) | **0.581 ± 0.071 (0.441, 0.719)** |
| Year | 0.036 ± 0.110 (-0.196, 0.249) | -0.005 ± 0.051 (-0.106, 0.095) |
| Territory-Level Relative-NDVI | 0.070 ± 0.087 (-0.097, 0.241) | 0.013 ± 0.035 (-0.056, 0.082) |
| Population-Level NDVI | **-0.416 ± 0.105 (-0.636, -0.217)** | **0.152 ± 0.048 (0.056, 0.249)** |
| Territory Size | -0.100 ± 0.085 (-0.269, 0.061) | 0.017 ± 0.031 (-0.046, 0.080) |
| Mean Vegetation Height | -0.066 ± 0.089 (-0.243, 0.103) | -0.012 ± 0.038 (-0.086, 0.064) |
| Female Age  (Relative to 1 Year Old) |  |  |
| *2+ Years Old* | **-0.571 ± 0.141 (-0.852, -0.291)** | **0.230 ± 0.064 (0.113, 0.354)** |
| Number of Helpers  (Relative to 0 Helpers) |  |  |
| *1 Helper* | -0.313 ± 0.162 (-0.635, 0.010) | **0.199 ± 0.063 (0.075, 0.319)** |
| *2+ Helpers* | **-1.035 ± 0.232 (-1.506, -0.563)** | **0.160 ± 0.076 (0.013, 0.309)** |
| Random Effects | $\sqrt{\text{Variance}}$ ± SD (95% CI) | $\sqrt{\text{Variance}}$ ± SD (95% CI) |
| Year | 0.353 ± 0.118 (0.128, 0.598) | 0.179 ± 0.048 (0.094 0.283) |
|  | (n = 27) | |
| Female ID | 0.267 ± 0.179 (0.014, 0.647) | 0.065 ± 0.044 (0.003, 0.163) |
|  | (n = 585) | |
| Territory ID | 0.180 ± 0.122 (0.009, 0.452) | 0.054 ± 0.039 (0.002, 0.143) |
|  | (n = 227) | |
| Spatial Correlation | 0.644 ± 0.349 (0.041, 1.314) | 0.215 ± 0.096 (0.027, 0.403) |
|  | n = 1187 samples | |

***Table S7:*** *Spatial and temporal variation in NDVI values during our study period.*

| **Parameter** | **Mean ± SD** | **Range** | **N** |
| --- | --- | --- | --- |
| Monthly Territory-Level NDVI | 0.60 ± 0.08 | 0.19–0.81 | 15745 territory-month-years |
| Monthly Population-Level NDVI | 0.61 ± 0.06 | 0.44–0.74 | 260 month-years |
| Spring–Summer Territory-Level NDVI | 0.58 ± 0.06 | 0.28–0.76 | 1703 territory-years |
| Spring–Summer Population-Level NDVI | 0.58 ± 0.03 | 0.53–0.65 | 27 years |
| Autumn–Winter Territory-Level NDVI | 0.64 ± 0.06 | 0.28–0.78 | 1604 territory-years |
| Autumn–Winter Population-Level NDVI | 0.65 ± 0.03 | 0.59–0.71 | 26 years |
| Annual Territory-Level NDVI | 0.60 ± 0.06 | 0.32–0.77 | 1703 territory-years |
| Annual Population-Level NDVI | 0.61 ± 0.03 | 0.55–0.68 | 27 years |

***Table S8:*** *Summaries of the eight Bayesian Generalised Additive Models (GAMs; with Gaussian-error distributions) used to test whether any of the three weather parameters or the population-level NDVI had changed directionally across the study period during spring–summer and autumn–winter.*

| **Model** | **Time Period** | **Environmental Response Parameter** | **Smooth Parameter**  Estimate ± SD (95% CI) | **Intercept**  Estimate ± SD (95% CI) | **Year**  Estimate ± SD (95% CI) |
| --- | --- | --- | --- | --- | --- |
| (a) | Spring–Summer | Maximum Temperature | **0.106 ± 0.128 (0.002, 0.474)** | **24.630 ± 0.207 (24.230, 25.049)** | **-0.292 ± 0.097 (-0.482, -0.095)** |
| (b) | Spring–Summer | Minimum Temperature | **0.267 ± 0.176 (0.020, 0.644)** | **10.093 ± 0.108 (9.885, 10.309)** | -0.065 ± 0.051 (-0.167, 0.040) |
| (c) | Spring–Summer | Rainfall | **10.649 ± 12.640 (0.271, 45.825)** | **396.346 ± 20.893 (356.980, 438.305)** | -4.585 ± 10.199 (-24.971, 15.542) |
| (d) | Spring–Summer | Population-Level NDVI | **0.003 ± 0.004 (0.000, 0.012)** | **0.584 ± 0.006 (0.572, 0.595)** | 0.004 ± 0.003 (-0.001, 0.010) |
| (e) | Autumn–Winter | Maximum Temperature | **0.071 ± 0.075 (0.003, 0.271)** | **15.399 ± 0.118 (15.171, 15.632)** | -0.095 ± 0.054 (-0.207, 0.010) |
| (f) | Autumn–Winter | Minimum Temperature | **0.083 ± 0.097 (0.003, 0.343)** | **2.443 ± 0.184 (2.080, 2.813)** | 0.042 ± 0.086 (-0.129, 0.205) |
| (g) | Autumn–Winter | Rainfall | **9.135 ± 10.290 (0.541, 37.176)** | **191.108 ± 13.705 (164.156, 218.112)** | 3.349 ± 6.403 (-9.678, 15.764) |
| (h) | Autumn–Winter | Population-Level NDVI | **0.009 ± 0.007 (0.001, 0.025)** | **0.649 ± 0.005 (0.639, 0.659)** | 0.003 ± 0.002 (-0.002, 0.008) |

*Note, there were no usable population-level NDVI data for the 2007 autumn–winter period (see main text for further details). Thus, model (h) was based on 26 rather than 27 years of data. The parameter estimates are presented as posterior means ± standard deviation (SD) and 95% credible intervals (CI). All explanatory parameters were mean standardised for analysis. Fixed effect parameters for which the 95% CI do not overlap zero are highlighted in bold.*

***Table S9:*** *Summaries of the two top-ranked Bayesian linear regression models of population-level NDVI when testing for weather effects.*

| **Spring–Summer** |  |
| --- | --- |
| **Parameters** | **Population-Level NDVI** |
| Fixed Effects | Estimate ± SD (95% CI) |
| Intercept | **0.586 ± 0.004 (0.578, 0.594)** |
| Year | **0.022 ± 0.005 (0.013, 0.031)** |
| Rainfall (Lagged) | **0.016 ± 0.004 (0.009, 0.024)** |
| Maximum Temperature | **-0.017 ± 0.005 (-0.027, -0.008)** |
| Rainfall (Lagged): Maximum Temperature | 0.008 ± 0.004 (-0.000, 0.016) |
| **Autumn–Winter** |  |
| **Parameters** | **Population-Level NDVI** |
| Fixed Effects | Estimate ± SD (95% CI) |
| Intercept | **0.658 ± 0.008 (0.643, 0.673)** |
| Year | **0.025 ± 0.011 (0.004, 0.048)** |
| Rainfall (Lagged) | -0.011 ± 0.013 (-0.036, 0.015) |
| Maximum Temperature (Lagged) | **-0.030 ± 0.015 (-0.061, -0.002)** |
| Rainfall (Lagged): Maximum Temperature (Lagged) | 0.014 ± 0.007 (-0.001, 0.028) |

*Note, there were no usable population-level NDVI data for the 2007 autumn–winter period (see main text for further details). Thus, this model was based on 26 rather than 27 years of data. The parameter estimates are presented as posterior means ± standard deviation (SD) and 95% credible intervals (CI). All explanatory parameters were mean standardised for analysis. Fixed effect parameters for which the 95% CI do not overlap zero are highlighted in bold. The term ‘lagged’ demonstrates that weather data were from the preceding season.*

***Figure S1:*** *Randomisation tests for top-ranked models. We considered a given top model to be reliable if its observed ELPD _LOO_ value (dashed black line) lay outside the distribution of all ELPD _LOO_ values from the 100 models using randomised data (i.e., PELPD _LOO_ <0.01).*


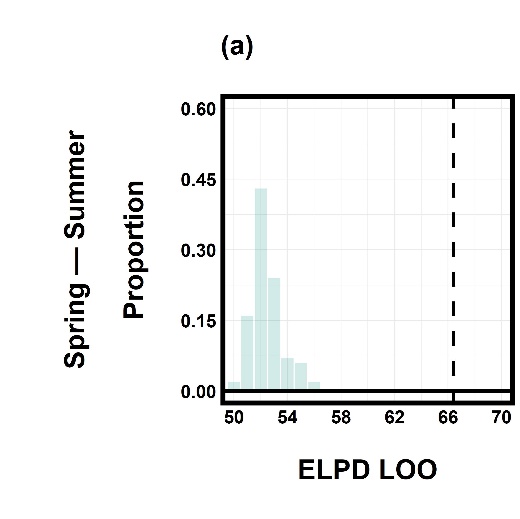

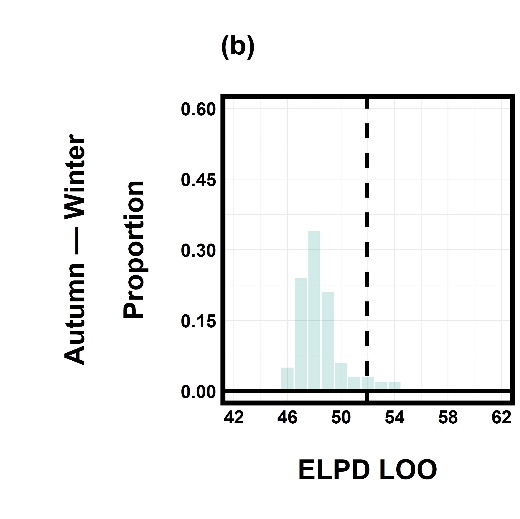


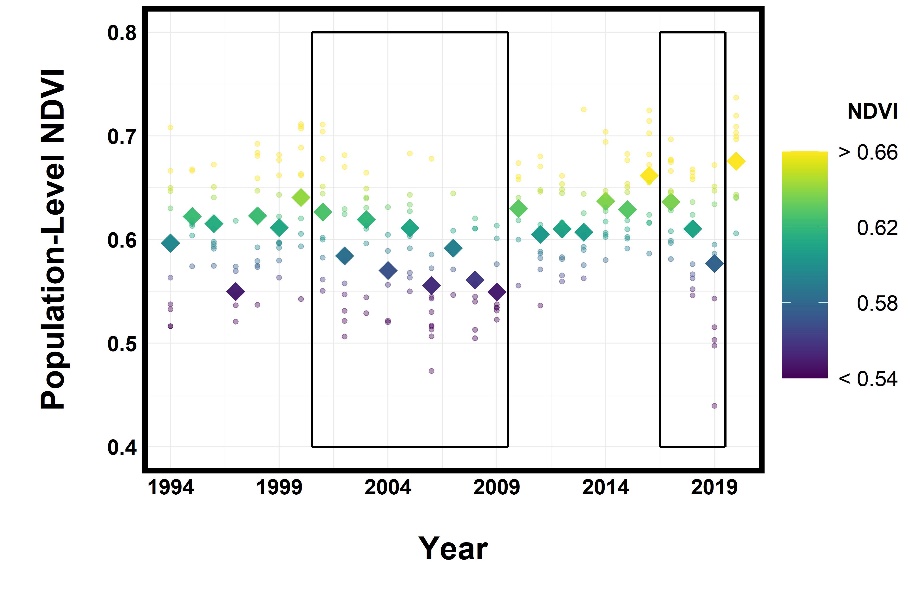


***Figure S2:*** *Variation in NDVI over time. The diamonds represent the annual population-level NDVI for each year throughout the study, while the points indicate the monthly population-level NDVI values within each year. The boxes highlight the two drought periods observed within the study area.*

**Supplementary methods**

*Identifying effects of weather on population-level NDVI*

Prior to evaluating the effects of weather conditions on population-level NDVI, we first established baseline models that accounted for non-weather-related sources of variation (e.g. solar radiation exposure and photoperiod length). We constructed four Bayesian linear regression models of population-level NDVI for each of the spring–summer and autumn–winter periods. These baseline models did not include any weather parameters, but instead separately incorporated linear or non-linear (i.e., quadratic, cubic, or quartic) fixed effects of year (fitted as a continuous covariate).

To determine the year effect that best fit the population-level NDVI data, we compared the predictive performance of these models using Bayesian leave-one-out expected log predictive density (ELPD _LOO_; Vehtari et al., 2017, 2020). Model comparisons were implemented using the *loo* function in the R package ‘loo’ (v.2.4.1; Vehtari et al., 2020). This approach repeatedly refits each model while excluding one observation at a time and computes the log likelihood of posterior predictive distributions for the omitted data point (Vehtari et al., 2017). These log likelihoods are then averaged across all observations to obtain the ELPD _LOO_ estimate.

As with other information criteria, such as Akaike’s information criterion (AIC; Akaike, 1974) or the widely applicable information criterion (WAIC; Watanabe, 2013, 2021; Watanabe & Opper, 2010), ELPD _LOO_ provides a measure of relative predictive performance, albeit on a different scale (Gelman et al., 2014). Models with higher ELPD _LOO_ values are considered to have better predictive performance. Typically, models are considered to be distinct if the difference in ELPD _LOO_ between them (ΔELPD _LOO_) is greater than 4 (Bürkner *et al.*, 2020; Sivula *et al.*, 2020; Vehtari *et al.*, 2017). The results from our comparison indicated that all models in each analysis demonstrated similar predictive performance (i.e., ΔELPD _LOO_ <4; Table S3). Therefore, we selected the most parsimonious models, which included only a linear effect of year, as the initial baseline for our spring–summer and autumn–winter population-level NDVI analyses.

*Calculating mean vegetation height*

Because superb fairy-wrens primarily forage in understory vegetation (Rowley, 1965; Rowley & Russell, 1997), we initially included *mean vegetation height* as a fixed effect in both mortality and breeding success analyses to account for potential canopy influences on NDVI.

Estimates of *mean vegetation height* were derived from airborne laser scanning (ALS) data collected across the study area between 21 May 2015 and 5 April 2016 by the Australian Capital Territory Government’s Environment, Planning and Sustainable Development Directorate ([www.planning.act.gov.au](http://www.planning.act.gov.au)). Details of the ALS survey are provided in Turner *et al.* (2023). The ALS data were pre-processed by the vendor and included point classifications for ground, buildings, water, vegetation, and noise.

Data processing was conducted in R (v.4.0.5; R Core Team, 2021) using the package ‘lidR’ (v.3.1.3; Roussel *et al.*, 2020). *Mean vegetation height* was calculated for each territory ID following four steps. First, the ALS point cloud was normalised by subtracting ground point elevations from non-ground point elevations. Second, points classified as ground, buildings, water, or noise were removed, retaining only vegetation points. Third, the remaining vegetation points were spatially subset by each territory ID. Finally, *mean vegetation height* for each territory was calculated as the mean height of all vegetation points within that territory.

**REFERENCES**

Akaike, H. (1974). A new look at the statistical model identification. *IEEE* *Transactions on Automatic Control*, *19*, 716–723.

Bürkner, P.C., Gabry, J., & Vehtari, A. (2020). Approximate leave-future-out cross-validation for Bayesian time series models. *Journal of Statistical Computation and Simulation*, *90*, 2499–2523.

R Core Team. (2021). *R: A language and environment for statistical computing*. Vienna, Austria: R Foundation for Statistical Computing.

Roussel, J.R., Auty, D., Coops, N.C., Tompalski, P., Goodbody, T.R.H., Sánchez Meador, A., Bourdon, J.F., De Boissieu, F. & Achim, A. (2020). lidR: An R package for analysis of Airborne Laser Scanning (ALS) data. *Remote Sensing of Environment, 251*, 112061.

Rowley, I. (1965). The life history of the superb blue wren *Malurus cyaneus*. *Emu-Austral Ornithology,* *64*, 251–297.

Rowley, I., & Russell, E.M. (1997). *Fairy-wrens and grasswrens: Maluridae*. Oxford UK: University Press.

Sivula, T., Magnusson, M., Matamoros, A.A., & Vehtari, A. (2020). Uncertainty in Bayesian leave-one-out cross-validation based model comparison. *arXiv*. Available from: https://doi.org/10.48550/arXiv.2008.10296.

Turner, R.S., Lasne, O.J.D., Youngentob, K.N., Shokirov, S., Osmond, H.L., & Kruuk, L.E.B. (2023). Use of Airborne Laser Scanning to assess effects of understorey vegetation structure on nest-site selection and breeding performance in an Australian passerine bird. *Remote Sensing in Ecology and Conservation, 9,* 787–802.

Vehtari, A., Gabry, J., Magnusson, M., Yao, Y., Bürkner, P., Paananen, T., & Gelman, A. (2020). loo: Efficient leave-one-out cross-validation and WAIC for Bayesian models. R package version 2.4.1. Available from: https://mc-stan.org/loo.

Vehtari, A., Gelman, A., & Gabry, J. (2017). Practical Bayesian model evaluation using leave-one-out cross-validation and WAIC. *Statistics and Computing, 27,* 1413–1432.

Watanabe, S. (2013). A widely applicable Bayesian information criterion. *The Journal of Machine Learning Research*, *14*, 867–897.

Watanabe, S. (2021). WAIC and WBIC for mixture models. *Behaviormetrika*, *48*, 5–21.

Watanabe, S., & Opper, M. (2010). Asymptotic equivalence of Bayes cross validation and widely applicable information criterion in singular learning theory. *Journal of machine learning research*, *11*, 3571–3594.
